# Link between the EZH2 noncanonical pathway and microtubule organization center polarization during early T lymphopoiesis

**DOI:** 10.1038/s41598-022-07684-5

**Published:** 2022-03-07

**Authors:** Frederique Deshayes, Magali Fradet, Sandra Kaminski, Mireille Viguier, Jean-Pol Frippiat, Stephanie Ghislin

**Affiliations:** 1 Université de Paris, CNRS, Unité de Biologie Fonctionnelle et Adaptative, Paris, France; 2 Université de Paris, CNRS, Institut Jacques Monod, 75006 Paris, France; 3 EA 7300, Stress Immunity Pathogens Laboratory, Faculty of Medicine, Lorraine University, 9 avenue de la forêt de Haye, 54500 Vandœuvre-lès-Nancy, France

**Keywords:** Cytoskeleton, Centrosome, T cells, T-cell receptor, Lymphocyte differentiation

## Abstract

EZH2 plays an essential role at the β-selection checkpoint of T lymphopoiesis by regulating histone H3 lysine 27 trimethylation (H3K27me3) via its canonical mode of action. Increasing data suggest that EZH2 could also regulate other cellular functions, such as cytoskeletal reorganization, via its noncanonical pathway. Consequently, we investigated whether the EZH2 noncanonical pathway could be involved in early T-cell maturation, which requires cell polarization. We observed that EZH2 localization is tightly regulated during the early stages of T-cell development and that EZH2 relocalizes in the nucleus of double-negative thymocytes enduring TCRβ recombination and β-selection processes. Furthermore, we observed that EZH2 and EED, but not Suz12, colocalize with the microtubule organization center (MTOC), which might prevent its inappropriate polarization in double negative cells. In accordance with these results, we evidenced the existence of direct or indirect interaction between EED and α-tubulin. Taken together, these results suggest that the EZH2 noncanonical pathway, in association with EED, is involved in the early stages of T-cell maturation.

## Introduction

During T-cell development, thymocytes undergo fine-tuned maturation steps, leading to the production of functional CD4 + or CD8 + T cells. These different steps are identified through the expression of specific cell surface markers. Eight different stages can be defined: four early double negative (DN) stages called DN1 (CD44 + CD25 −), DN2 (CD44 + CD25 +), DN3 (CD44-CD25 +) and DN4 (CD44-CD25−) and four later stages called intermediate single positive CD8 (ISP8) (CD4-CD8 + CD3-TCR-), double positive (DP) (CD4 + CD8 +), single positive CD4 (SP4) (CD4 + CD8 − CD3 + TCR +) and single positive CD8 (SP8) (CD4-CD8 + CD3 + TCR +).

During the DN2 to DN3 transition, TCRβ gene segments rearrange to produce a TCRβ chain that associates with the pTCRα chain and CD3 to form a pre-TCR-CD3 complex (pTCR-CD3). The functionality of this pTCR-CD3 complex is then tested through β-selection occurring at the DN3 stage, hence allowing the elimination of T-cells expressing nonfunctional TCRβ. Defects in this process can lead to severe pathologies such as malignant transformation of early T cells^1^.

Different actors of β-selection have been identified, among which some are involved in chromatin structure regulation. One of these actors is the histone methyltransferase EZH2^2,3^. EZH2 is part of Polycomb Repressive Complex 2 (PRC2) with Suz12, EED and RbAp46/48. Through its canonical pathway, EZH2 is responsible for histone H3 lysine 27 mono-, di- and trimethylation (H3K27me1, 2 or 3)^4^. At the β-selection checkpoint, EZH2 regulates Cdkn2a through methylation of its promoter, which prevents p53 stabilization; in this context, the absence of EZH2 induces blockade of T cell differentiation at the DN3 stage^5^. Interestingly, growing evidence shows that EZH2 can also regulate different cellular processes^6,7^, such as actin reorganization, through a noncanonical pathway, implying the methylation of nonhistone proteins. Indeed, in mature T cells, EZH2 is directly involved in TCR signaling by regulating actin polymerization^3^.

Consequently, we evaluated the role of the EZH2 noncanonical pathway during β-selection in T-cell development. We studied EZH2 subcellular localization as well as EZH2 and H3K27me3 levels in thymocytes. Interestingly, while no correlation was found between EZH2 expression and canonical activity, we established that EZH2 subcellular localization was tightly orchestrated during the early stages of T-cell differentiation. More importantly, we observed that EZH2 and EED localized in the cytoplasm, where they could act through a noncanonical pathway. Further analysis allowed us to detect for the first time that EZH2 and EED colocalize with MTOC and inhibit its polarization in the absence of pTCR-CD3. Co-immunoprecipitation experiments suggest an interaction between EED (and potentially EZH2) and the tubulin network. Altogether, these results lead us to propose a novel link between the EZH2/EED noncanonical pathway and MTOC polarization during early T lymphopoiesis.

## Results

### EZH2 localization is tightly regulated during early T-cell development

Since EZH2 has a noncanonical cytoplasmic function in mature T cells^3^, we examined the variation in EZH2 subcellular localization during T lymphopoiesis. To this end, we developed a specific protocol to perform flow cell imaging using an Imagestream × multispectral imaging flow cytometer. As we observed two CD44 staining intensities in the DN1 subpopulation, we analyzed both populations separately. Hence, five subpopulations of EZH2-expressing double-negative cells could be defined: DN1 CD44_high_ (CD44^high^/CD25^−^), DN1 CD44_low_ (CD44^low^/CD25^−^), DN2 (CD44^low^/CD25^+^), DN3 (CD44^-^/CD25^+^) and DN4 (CD44^−^ CD25^−^) (Fig. S1). Later, EZH2-expressing maturation stages were defined as described in the introduction (Fig. S1A, B, C and E).

**Figure 1:**
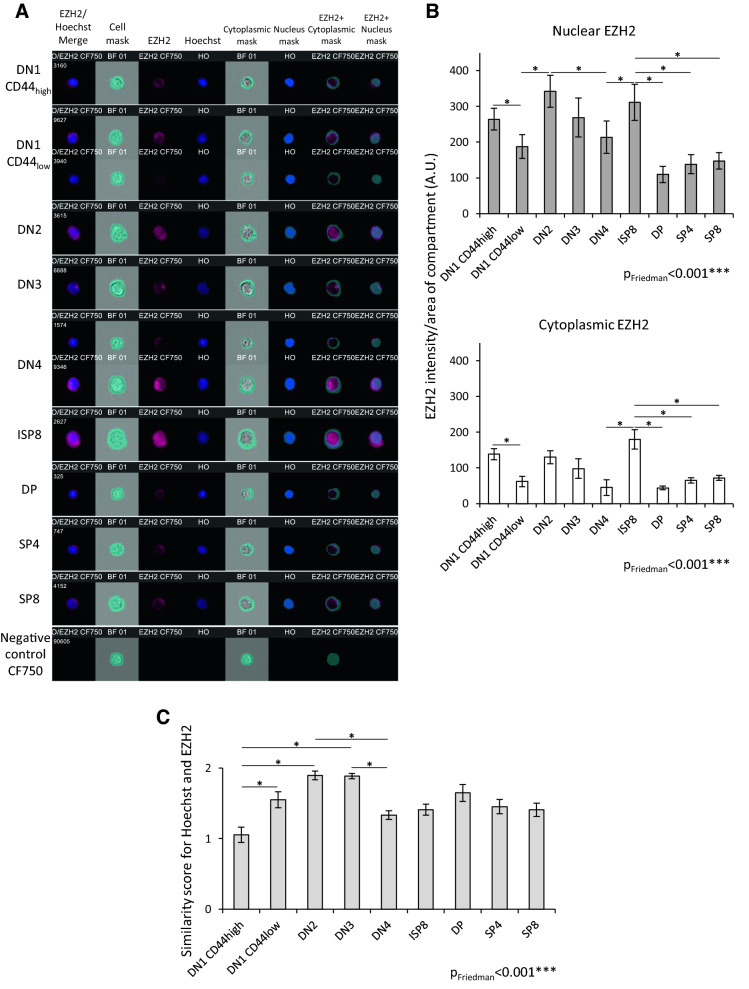
EZH2 subcellular localization changes during T lymphopoiesis as revealed by flow cytometry imaging. Thymocytes were identified using CD4, CD8, CD3, TCR, CD25 and CD44 staining. (**A**) Representative EZH2 staining at different stages of T lymphopoiesis. A mask representing the nucleus was defined using Hoechst 33,342 staining and the morphology mask. The whole cell mask was defined using brightfield and object masks. Another mask representing the cytoplasmic area was defined by subtracting the nucleus mask from the whole cell mask. Masks are indicated in turquoise blue. For each subpopulation, merged EZH2/Hoechst staining, whole cell mask, EZH2 staining, Hoechst staining, cytoplasmic and nuclear masks, EZH2 staining within the cytoplasmic mask and EZH2 staining within the nuclear mask are presented. For each stage, one representative of the observed cells is shown. When, two subpopulations were observed in a stage, two images are shown. Number on the left of each image corresponds to the number of cells attributed to the event by the software during the acquisition process. (**B**) EZH2 quantity in nuclear (upper panel) and cytoplasmic (lower panel) compartments was defined as EZH2 intensity divided by compartment area. (**C**) EZH2/Hoechst colocalization estimated using the similarity score. Data are means ± SD of 10 mice. Friedman test was performed followed with its post hoc test. Friedman p values are indicated for each analysis. * indicates significant differences between subpopulations revealed by the post hoc pairwise Wilcoxon rank sum test.

EZH2 was detected in the nucleus and cytoplasm (Fig. 1A) at each stage, but EZH2 concentration was always higher in the nucleus than in the cytoplasm (Fig. 1B). Moreover, we observed concentration variations in the nucleus and cytoplasm between stages (Fig. 1B) that could be due to EZH2 expression changes and/or shuttling of EZH2 between both compartments, but both phenomena could not be discriminated using intensity value (Fig. S1D). As a consequence, we decided to test if EZH2 shuttling occurs between the cytoplasm and nucleus during differentiation. For this purpose, we calculated similarity scores, as described by George et al.^8^, to analyze localization independently of expression level variation. The higher this score is, the more EZH2 is localized in the nucleus (Hoechst staining). This analysis revealed a 1.8-fold increase in the score between DN1 CD44_high_ and DN3 stages and a 0.7-fold decrease between DN3 and DN4 (Fig. 1C). These results advocate for a relocalization of EZH2 in the nucleus during TCRβ recombination and β-selection processes (stages DN2-DN3). In contrast, EZH2 cellular localization was stable in the following stages (from ISP8 to SP4/SP8) (Fig. 1C).

Thus, EZH2 localization is tightly regulated during early stages of T-cell development, suggesting important functions at these stages. Furthermore, as EZH2 is localized in both the cytoplasm and the nucleus, we hypothesized that it might act through its canonical and/or noncanonical pathways during early T lymphopoiesis.

### EZH2 expression and H3K27me3 levels do not correlate during T lymphopoiesis

**Figure 2:**
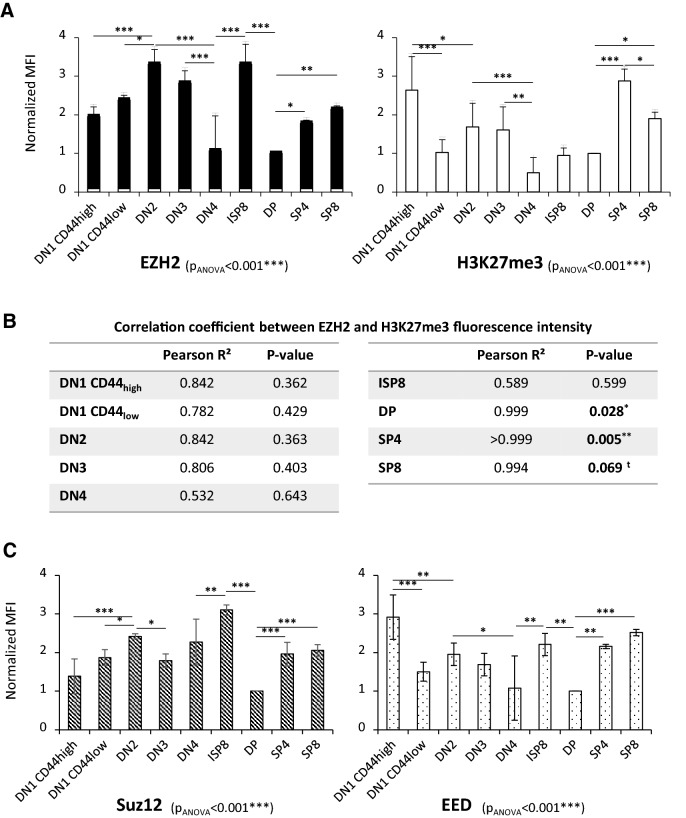
The EZH2 expression level is tightly regulated during T lymphopoiesis and is not correlated with H3K27 trimethylation. EZH2, H3K27me3, EED and Suz12 levels were evaluated at each stages of T lymphopoiesis by flow cytometry. (**A**) EZH2 (left panel) and H3K27me3 (right panel) median fluorescence intensity (MFI) variation during T lymphopoiesis. The MFI for each subpopulation was normalized to the MFI of DP. Data are mean ± SD of 3 independent experiments. ANOVA tests were performed followed by post hoc PLSD Fisher tests. *P < 0.05, **P < 0.01, ***P < 0.001. (**B**) Absence of correlation between EZH2 and H3K27me3 levels in double-negative stages and ISP8 stage. For each stage, the median EZH2 fluorescence was plotted against the median H3K27me3 fluorescence. The Pearson correlation coefficient (Pearson R^2^) was calculated, and the bilateral Pearson test was performed and is indicated in the table. ^t^*P* < 0.07*, *P* < 0.05, **P < 0.01. (**C**) Suz12 (left panel) and EED (right panel) MFI variation during T lymphopoiesis. The MFI for each subpopulation was normalized to the MFI of DP. Data are mean ± SD of 3 independent experiments. ANOVA tests were performed followed by post hoc PLSD Fisher tests. *P < 0.05, **P < 0.01, ***P < 0.001.

To determine how EZH2 acts during early T lymphopoiesis, we measured by flow cytometry EZH2 expression and H3K27 trimethylation levels in all stages of T cell development. Our analysis showed that the levels of EZH2 and H3K27me3 were homogeneous in each subpopulation, except in DN4 and ISP8 thymocytes (Fig. S2A). After normalization to DP expression levels (stage chosen because of the same level of EZH2 and H3K27me3 at this stage), no correlation between EZH2 and H3K27me3 levels was found during early T lymphopoiesis up to the ISP8 stage (Figs. 2A and B, S2A and B). As, a correlation between EZH2 expression and H3K27me3 level was not observed, this suggest the existence of an EZH2 non-canonical activity. Because EZH2 activity is also dependent on its partners EED and Suz12, we studied their expression during T lymphopoiesis. As shown in Figs. 2C, S2C and S2D, EED and Suz12 levels seem to be finely tuned during differentiation. Interestingly, in DN stages, the pattern of EED variation was more similar to the one of H3K27me3 than to EZH2 changes. Inversely, Suz12 pattern was more similar to EZH2 than to H3K27me3 patterns. Concerning DP and SP4/SP8 stages, EZH2, EED and Suz12 presented the same variation profile. Altogether these results suggested the existence of a specific regulation of PRC2 complex members expression during T lymphopoiesis, specifically at DN stages.

The absence of correlation at DN stages between EZH2 expression and H3K27me3 levels suggests that EZH2 could act through a noncanonical pathway in addition to the previously shown canonical pathway^5^.

### EZH2 and EED colocalize with tubulin network

**Figure 3:**
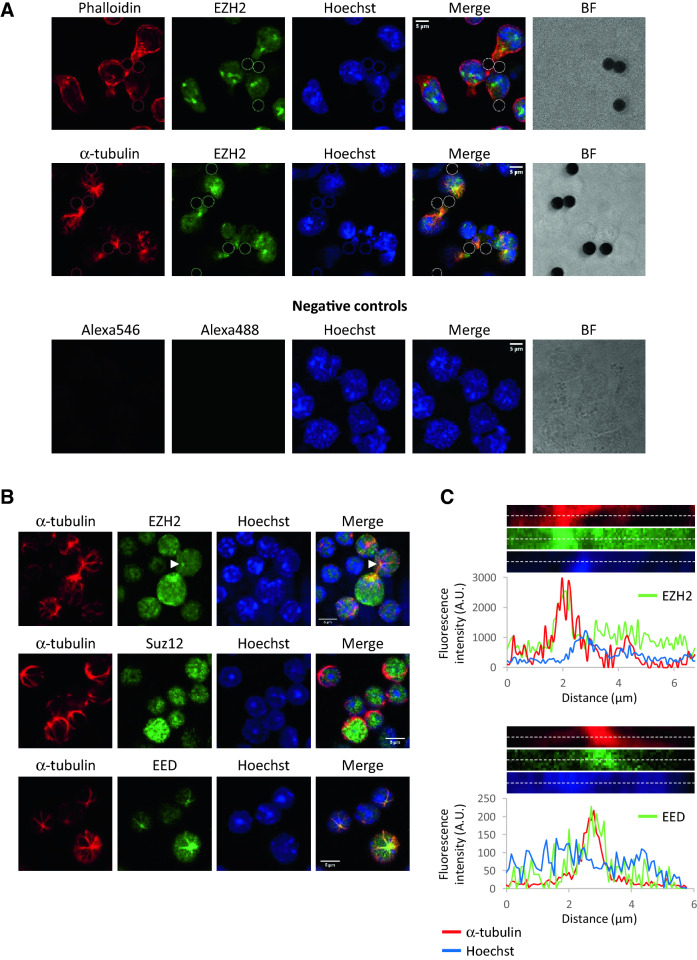
EZH2 and EED colocalize with α-tubulin. (**A**) The SCB29 cell line was stimulated with Dynabeads™ Mouse T-activator CD3/CD28 for 30 min. Localization of EZH2 (green) and F-actin (phalloidin, red) or α-tubulin (red) was analyzed. Bead localization was determined using brightfield (BF) and reported in each channel. Negative controls were performed using secondary antibodies. Scale bar 2 µm. (**B**) Costaining of EZH2, EED or Suz12 (green), α-tubulin (red) and Hoechst (blue) in murine thymocytes. The arrow indicates the cell analyzed in (**C**). Scale bar 5 µm. (**C**) Maximum fluorescence of EZH2 or EED and α-tubulin (corresponding to the centrosome) colocalize in thymocytes. For one representative cell, the histogram indicates the fluorescence intensity measured for EZH2 or EED, α-tubulin and the Hoechst channel in the center of the tubulin aster region. Dotted lines in pictures above histograms indicate analyzed regions.

Since actin cytoskeleton and microtubule reorganization are known to be essential for T cell development, especially at DN stages^9–11^, we decided to focus on EZH2 and polymerized actin or α-tubulin colocalization after pTCR-CD3 activation. After validating the specificity of the anti-EZH2 antibody by transfecting the EL4 cell line with a specific EZH2 siRNA (Fig. S3A and B), we performed immunofluorescence with the DN3 SCB29 cell line, which expresses a functional pTCR, stimulated for 30 min with CD3/CD28 beads. We did not observe any specific colocalization of EZH2 with F-actin (phalloidin) (Fig. 3A, upper panel). However, EZH2 colocalized with α-tubulin to form a spot prolonged by filamentous structures that matched the maximum fluorescence of α-tubulin, probably corresponding to the MTOC region (Fig. 3A, lower panel). To ensure the relevance of this last observation, we performed EZH2 and α-tubulin staining in primary mouse thymocytes in the absence of stimulation with two different anti-EZH2 antibodies. The same results were obtained, confirming the existence of this colocalization between EZH2, the tubulin network and probably the MTOC region (Figs. 3B,C and S3C) with or without immature T cell activation. Then, we asked if EZH2 partners EED and Suz12 also colocalize with the tubulin network in thymocytes. To this end, immunofluorescences were performed with EED or Suz12 and α-tubulin antibodies. Interestingly, as for EZH2, we observed heterogenous staining for both EED and Suz12. Suz12 seems to be exclusively localized in the nucleus while EED seems to be localized in both the cytoplasm, where it colocalizes with tubulin network (Fig. 3B,C), and the nucleus. These results led us to believe that only EZH2 and EED colocalize with the tubulin network and probably the MTOC region.

### EED interacts with α-tubulin

**Figure 4:**
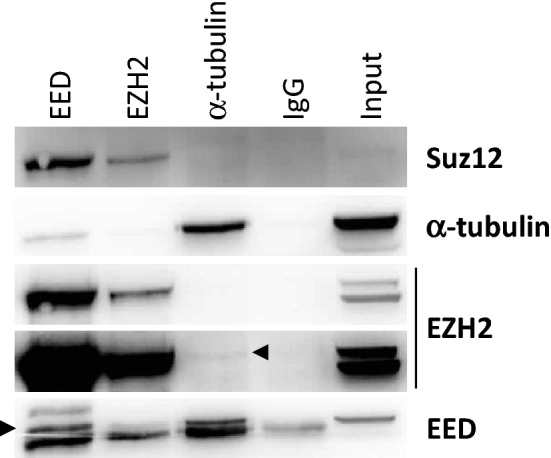
EED interacts with α-tubulin. Co-immunoprecipitation was performed using the SCIET27 cell line. EED, EZH2, α-tubulin or IgG used as negative control were immunoprecipitated and Suz12, EED, EZH2 and α-tubulin were detected by western blotting. For EZH2, two different expositions are shown to evidence low intensity bands. A representative western blot of the 3 performed is shown.

To evaluate if EED and/or EZH2 interact with the tubulin network, we performed co-immunoprecipitation experiments in SCIET27 cells. We immunoprecipitated EED, EZH2 or α-tubulin and evaluated co-immunoprecipitation of Suz12, EED, EZH2 and α-tubulin. As shown in Fig. 4, immunoprecipitation of EED led to the co-precipitation of Suz12, EZH2 and α-tubulin. Immunoprecipitation of α-tubulin precipitated EED and a very small amount of EZH2, while Suz12 did not precipitate. These results strongly suggest the existence of interactions between α-tubulin and EED and probably EZH2 and α-tubulin (directly or indirectly through EED).

### EZH2/EED complex participates for regulation of MTOC polarization

Because β-selection is known to induce the polarization of the MTOC, which is associated with asymmetric division during DN3 transition^11^, we investigated the potential role of EZH2/EED in MTOC polarization during β-selection by comparing the DN3 SCB29 cell line expressing a functional pTCR and the DN2 SCIET27 cell line deficient for pTCR.

**Figure 5:**
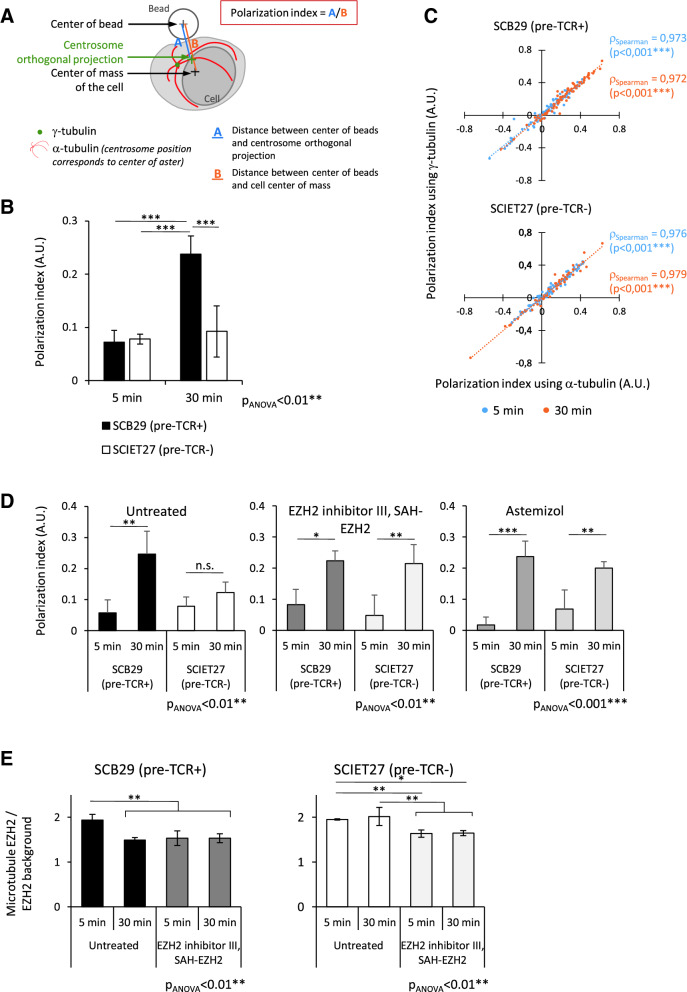
EZH2/EED complex regulates MTOC polarization in the absence of pre-TCR. (**A**) Schematic representation of the polarization index calculation. MTOC position was determined using γ-tubulin staining (green point) or center of a-tubulin aster (red). The gray cross corresponds to the bead center (B_C_), and the black cross corresponds to the cell center of mass (Cell_CM_). The position of MTOC was orthogonally projected on the B_C_-Cell_CM_ vector, and its position is indicated by a green cross. The polarization index was calculated by dividing the length of the B_C_-MTOC projection vector (blue A) by the length of the B_C_-Cell_CM_ vector (orange B). (**B**) SCB29 and SCIET27 were stimulated with Dynabeads™ Mouse T-activator CD3/CD28 for 5 or 30 min. MTOC position was determined using γ-tubulin staining (green), and bead localization was determined using brightfield microscopy. The MTOC polarization index was calculated as described in (A) for the 35–95 cell-bead couple at each time point. (**C**) α-tubulin can be used to calculate the polarization index. SCB29 and SCIET27 cell lines were stimulated as in (B), and immunofluorescence was performed. Both α-tubulin and γ-tubulin were costained. For each cell-bead couple, the polarization index was calculated using γ-tubulin and α-tubulin independently. Then, the correlation between both polarization indices was calculated using the Spearman correlation test for each parameter for three independent experiments. The dot plot represents the polarization index calculated using γ-tubulin staining against the polarization index calculated using α-tubulin staining. Blue points correspond to the 5-min time point, and orange points correspond to the 30-min time point for each cell-bead couple. It corresponds to one representative experiment. The Spearman correlation coefficient (ρ_Spearman_) was calculated, and the bilateral Spearman test was performed and is indicated on the plots. ***P < 0.001. (**D**, **E**) SCB29 and SCIET27 cell lines were pretreated for a minimum of 1 h30 with EZH2 inhibitor III EZH2 SAH (5 µM) or astemizole (5 µM) and then stimulated with Dynabeads™ Mouse T-activator CD3/CD28 for 5 or 30 min. Then, immunofluorescence was performed, and α-tubulin was stained to evaluate the MTOC position. (**D**) MTOC polarization indices were calculated for each cell-bead couple. (**E**) For each cell-bead couple, the mean EZH2 staining intensity at microtubules and MTOC were measured and normalized over the mean EZH2 staining intensity in the background. The results are deduced from the analysis of 35 to 95 bead-cell couples per time point. For (**B**, **D** and **E**), histograms represent the mean of three independent experiments ± SD, and ANOVA tests were performed followed by post hoc PLSD Fisher tests. *P < 0.05, **P < 0.01, ***P < 0.001, n.s.: nonsignificant.

We first wondered whether pTCR-CD3 activation could induce MTOC polarization. We evaluated MTOC polarization by calculating the polarization index of MTOCs using γ-tubulin staining, as explained in Fig. 5A. Both SCB29 and SCIET27 cell lines were activated for 5 and 30 min with Dynabeads™ CD3/CD28. Immunofluorescence analyses shown in Fig. 5B show that MTOCs polarized to beads after 30 min of stimulation exclusively in the SCB29 cell line, as we observed a significant 3.3-fold increase in the polarization index between the 5- and 30-min time points, evidencing that pTCR activation could induce cell polarization.

We then asked whether EZH2/EED contributes to MTOC polarization regulation and whether EZH2 localization along microtubules was modified during this process. We chose to study EZH2 localization as it presents an enzymatic activity unlike EED. We thus simultaneously followed MTOC positioning and EZH2 localization along the microtubule network in the absence or presence of an EZH2/EED inhibitor. We first validated the usage of α-tubulin staining to estimate MTOC position using co-staining of α- and γ-tubulin in both cell lines. We observed that γ-tubulin was always localized at the center of the aster formed by the microtubule network after 5 and 30 min of stimulation (Fig. S4A). Calculation of the polarization index using γ-tubulin or the center of the aster observed with α-tubulin staining significantly correlated independently of stimulation time or cell lines, as shown in Fig. 5C. The average difference between the polarization index calculated with γ-tubulin or α-tubulin was 0.046 ± 0.022, which was significantly lower (*P* < 0.01) than the average difference calculated between 5 and 30 min for SCB29 (0.14 ± 0.027). Considering these results, we validated the use of α-tubulin to evaluate MTOC position.

Then, both cell lines were treated during a total of 2 h with two different EZH2/EED inhibitors blocking the EZH2-EED interaction before pTCR-CD3 stimulation: EZH2 inhibitor III-SAH-EZH2^12^ and astemizole^13^. We chose this strategy, instead of siRNA silencing, to avoid affecting the epigenome of the cells and consequently to avoid the superposition of transcriptome modifications (canonical function of EZH2) with non-canonical EZH2 pathway inhibition as explained by Tripathi and colleagues^14^. We first calculated the polarization index under each condition for both cell lines. As shown in Fig. 5D, in the absence of inhibitor (left panel), only SCB29 polarized after 30 min of stimulation. Interestingly, SCB29 polarization capability was not affected by EZH2/EED inhibitors, while SCIET27 could polarize in these conditions despite the absence of pTCR (Fig. 5D middle and right panels). Indeed, for SCIET27, in the absence of inhibitor, no significant variation in the polarization index was observed between both time points, while a 4.5- and 2.9-fold increase was observed after 30 min of stimulation in the presence of the EZH2 inhibitor III-SAH-EZH2 and astemizole, respectively. Since the EZH2/EED complex is dissociated from the inhibitor, we could expect a change in EZH2 localization from the MTOC after stimulation or due to inhibitor activities. Indeed, we observed different EZH2 staining in our cells, as illustrated in Fig. S4B. To precisely measure EZH2 localization variations along microtubules and in the MTOC region, we developed a mathematical approach. Briefly, the mean EZH2 staining intensity along MTOCs and/or microtubules was determined. The obtained mean intensity values were normalized to the mean EZH2 intensity determined in three randomly chosen cytoplasmic regions that did not contain microtubules. Thus, if EZH2 delocalizes from MTOCs and microtubules, a decrease in this ratio will be observed, and conversely, if EZH2 is concentrated in MTOCs. Using this method, we noted that EZH2 was delocalized under untreated conditions after pTCR activation (Fig. 5E, untreated SCB29). In the presence of the EZH2 inhibitor III-SAH-EZH2, we observed EZH2 delocalization in both cell lines at 5 and 30 min of stimulation (Fig. 5E).

These experiments have evidenced that MTOC polarization could be induced by pTCR-CD3 activation in an immature cell line, and that this polarization seems to be negatively regulated by the EZH2/EED complex in the absence of pTCR. Altogether, our results strongly suggest that EZH2/EED contributes to MTOC positioning regulation via its MTOC localization, which depends on the EZH2 and EED interaction.

## Discussion

PRC2 components have been suggested to be closely controlled to maintain hematopoiesis homeostasis^15–21^, but little is known concerning B and T lineages. EZH2 is essential for the differentiation of lymphocyte lineages, as its deletion induces T-cell differentiation arrest at the β-selection checkpoint^3,5^ and B-cell differentiation arrest at the pre-BCR selection step^2^, while its homolog EZH1 is only essential for B-cells development^21^. Interestingly, Suz12 KO showed that its lack affects both lineages, but unlike the EZH2 KO, all differentiation stages were affected. All these observations suggest a complex interplay of PRC2 components, but there are unfortunately no data concerning their expression variation along lymphocytes differentiation. Our flow cytometry studies suggest that EZH2, EED and Suz12 are tightly regulated during T lymphopoiesis but in different ways. Our data also suggest that EZH2 localization is finely tuned during T lymphopoiesis. In agreement with our results, Raaphorst and colleagues reported that progenitors and immature thymocytes express higher EZH2 levels than mature thymocytes^22^. They also observed a minor DN population negative for EZH2, which could correspond to the DN4 population, as these cells express EZH2 heterogeneously^22^. Furthermore, we observed that EZH2 localization is highly controlled in DN cells, with a maximum nuclear localization in DN2 and DN3 cells, which was not associated with an increase in H3K27me3. However, EZH2 is responsible for most H3K27 methylation in DN stages, as EZH2 KO DN cells present a significant decrease of H3K27me3 but no absence of this mark^3,5^. Moreover, EZH2 depletion was not rescued by EZH1 (a homolog of EZH2) whose expression was not affected by EZH2 depletion^5^. These results, combined with ours, lead us to propose that EZH2 acts via both its canonical and noncanonical pathways when localized in the nucleus of T-cells, as previously shown in other cell types^23–25^, and via its canonical pathway in immature^5^ and mature T-cells^26–28^.

In immune cells, EZH2 also has important functions in the cytoplasm. Indeed, it regulates the migration of neutrophils and dendritic cells via Talin methylation, an essential cytoskeletal protein^29^. EZH2 is also implicated in TCR signaling via the regulation of actin polymerization^3^ and the activation of the MAPK/Erk pathway^30^. The role of EZH2 has been suggested to be similar for pTCR signaling at the DN3 stage, but recent data suggest that it acts via its canonical pathway at this stage^5^. As our analysis of EZH2 expression and H3K27me3 levels by flow cytometry suggested that EZH2 could have a noncanonical function in DN stages, we decided to concentrate our investigation on DN2 and DN3 stages. The activation of the pTCR-CD3 complex in SCB29 did not induce EZH2 colocalization with F-actin or EZH2 relocalization at the point of pTCR activation unlike in mature T cell^3,5^. However, using a short time inhibition strategy, we propose, for the first time, that EZH2 not only localizes at the centrosome but, more importantly, regulates MTOC polarization during β-selection. To confirm this result, it would be interesting to construct conditional specific EZH2 KO DN2 cells as EZH2 is essential during development. However, such construction will affect the canonical pathway as well as cell transcriptome, making it difficult to discriminate the canonical and non-canonical role of EZH2 in DN2 cells. Indeed, the non-exhaustive analyses of RNAseq data from DN3 cells of EZH2 KO mice (Jacobsen et al.*)* showed an increase in at least 66 genes related to cytoskeleton (including 13 directly associated to microtubule and 2 associated to centrosome) and vesicular trafficking, and a decrease in at least 26 genes related to cytoskeleton (including 4 genes associated to microtubule). These gene expression changes could impact pTCR-CD3 complex regulation^14^. However, a long-term inhibition of EZH2 can complicate the interpretation of results as noted by Tripathi et al. (2021)^14^.

Furthermore, we made clear that CD3/CD28 stimulation induces MTOC polarization in a DN3 cell line expressing the pTCR. Accordingly, MTOC has been shown to polarize in DN3a thymocytes (DN3 that have not yet encountered β-selection) when interacting with stromal OP9-DL1 cells^11^, probably via pTCR-pMHC interaction, as demonstrated between DN3a and OP9-DL4 cells^31^. Additionally, the inhibition of EZH2/EED interaction allows MTOC polarization even in the absence of a pTCR in a DN2 cell line. This EZH2 MTOC regulation may be essential to drive normal T lymphopoiesis since CD28 and CD3ε are expressed in the absence of pTCR, and thus inappropriate stimulation of these chains could induce differentiation in DP without pTCR expression^32,33^. Such uncontrolled events would lead to major defects in T-cell development, as MTOC polarization occurs only at the DN3 stage^34^.

As EZH2 operates with EED and Suz12, their expression and localization were also analyzed. Interestingly, EED, but not Suz12, localizes at the centrosome, suggesting that EZH2 associates with EED, but not Suz12 along tubulin network. These observations are in accordance with co-immunoprecipitation results. Altogether these results suggest for the first time, a new function of the EZH2/EED complex in T cell development, independently of Suz12. In accordance with these results, EED was already shown, as EZH2, to act in cytoplasm^35,36^.

MTOC polarization has been associated with a reduction in actin nucleation in the centrosome after BCR stimulation^37^. Since EZH2 is known to regulate actin polymerization and EZH2 is partially delocalized from MTOC in our study, we hypothesized that centrosomal EZH2 activity leads to actin nucleation and avoids MTOC polarization in the absence of a complete pTCR signal. According to our results, this activity would depend on the EZH2-EED association. This new function of EZH2/EED complex in β-selection could not be observed using different EZH2 KO and notably after EZH2 rescue in EZH2-/- RAG1-/- experimentation^3,5^. Indeed, even if this function increases the viability of unwanted DN2 and DN3 by defective selection processes, the loss of EZH2, essential for cell survival, would induce the apoptosis of such cells^5^.

In conclusion, our results highlight a likely new role for EZH2 during early T lymphopoiesis for which molecular mechanisms will have to be deepened. In the future, it will necessary to investigate the putative role of this non-canonical EZH2 function in later selection steps of T lymphopoiesis. Moreover, it will be essential to determine whether this new function could also be associated with more diverse cellular functions, such as epigenetic modifications coupled to the cell cycle and cell differentiation or cell migration and homing.

## Methods

### Mice and cell lines

Seven-week-old C57BL6/J mice were purchased from Charles River Laboratories (Wilmington, MA USA). Animals were kept in the animal house of the Biology and Health Campus of the University of Lorraine (agreement number C54-547-30) until they were 9–12 weeks old. Animals were treated in accordance with the French Legislation and the Council Directive of the European Communities on the Protection of Animals Used for Experimental and Other Scientific Purposes (2010/63/UE). The Lorraine Ethics Committee on Animal Experimentation approved all mice work (CELMEA-66) and authors complied with ARRIVE guidelines. Animals were anesthetized using 5% isoflurane and then put to death by cervical dislocation before thymus collection.

SCIET27 and SCB29 were kindly provided by I. Screpanti (Laboratory of Molecular Pathology, Sapienza University of Rome) and I. Aifantis (New York University, School of Medicine). Cells were cultured in IMDM (Gibco, Thermo Fisher, Waltham, MA, USA) supplemented with penicillin streptomycin (Sigma–Aldrich, Saint-Quentin Fallavier, France) and 10% FCS (Gibco, Thermo Fisher, Waltham, MA USA) according to Aifantis et al. 2001^38,39^.

### Antibodies and reagents

Flow cytometry and flow cytometry cell imaging antibodies (anti-CD4 (RM4–5), anti-CD8a (53–6.7), anti-CD25 (3C7), anti-CD44 (IM7), anti-TCRβ (H57–597) and anti-CD3 (17A2)) and the respective isotype controls were purchased from Biolegend (Ozyme, Saint-Quentin-en-Yvelines, France). Secondary donkey anti-rabbit IgG-CF750 was purchased from Biotium (Ozyme, Saint Quentin Yvelines, France). Alexa Fluor 488 goat anti-rabbit IgG (H + L), Alexa Fluor 546 goat anti-rat IgG (H + L), Hoechst 33,342 and Dynabeads™ Mouse T-activator CD3/CD28 secondary antibodies were purchased from Thermo Fisher (Waltham, MA, USA). The secondary antibody DyLight® 488 goat anti-mouse IgG (H + L) was purchased from Bethyl (Montgomery, TX, USA). Anti-EZH2 antibody (AP2512d), used for immunofluorescence, flow cytometry, and western blot came from Abgent (CliniSciences, Nanterre, France). Anti-α-tubulin antibody (sc-53029) was purchased from Santa Cruz Biotechnology (CliniSciences, Nanterre, France). Anti-γ-tubulin (clone GTU-88) and anti-H3K27me3 antibodies (07–449) were purchased from Merck Millipore (St-Quentin-en-Yvelines, France). The EZH2 inhibitor astemizole was purchased from Euromedex (Souffelweyersheim, France), and the EZH2 inhibitor III SAH-EZH2 was purchased from Merck Millipore. Anti-EED antibody (ab4469), used for flow cytometry, immunofluorescence, co-immunoprecipitation and western blot, was purchased from Abcam (Cambridge, UK). For co-immunoprecipitation, anti-EZH2 antibody (07–689) came from Merck Millipore, anti-α-tubulin (PA5-29,444) came from Thermo Fisher and rabbit IgG control (C15410206) came from Diagenode (Liege, Belgium). Anti-Suz12 (D39F6), used for flow cytometry and western blot, came from Cell signaling (Danvers, MA, USA). Anti-α-tubulin (clone DM1A) used for western blot came from Merck Millipore. Goat anti-mouse and anti-rabbit IgG (H + L) secondary HRP antibodies and Dynabeads™ protein A were purchased from Thermo Fisher. Both siRNAs (Scramble, AM4611; EZH2 siRNA, AM16708, ID 157,425) came from Thermo Fisher.

### Flow cytometry

Thymuses from 9- to 12-week-old mice were dissociated in PBS-2% FCS and stained using different antibodies to identify subpopulations: DN1 (CD4^−^CD8^−^CD25^−^CD44^+^), DN2 (CD4^−^CD8^−^CD25^+^CD44^low^), DN3 (CD4^−^CD8^−^CD25^+^CD44^−^), DN4 (CD4^−^CD8^−^CD25^−^CD44^−^), ISP8 (CD4^−^CD8^+^CD25^−^CD44^−^CD3^low^TCRβ^low^), DP (CD4^+^CD8^+^), SP4 (CD4^+^CD8^−^CD3^high^TCRβ^high^) and SP8 (CD4^−^CD8^+^CD3^high^TCRβ^high^).

To identify ISP8, DP, SP4 and SP8 subpopulations, 5 × 10^5^ thymocytes were freshly stained for 30 min with the following combination of antibodies: anti-CD4-APC, anti-CD8a-PB, anti-CD3-PE-Cy7 and anti-TCRβ-PE. DN subpopulations were enriched by negative selection before flow cytometry using the EasySep™ Mouse streptavidin RapidSpheres™ isolation kit (Stem Cell Technology, Vancouver, Canada) coupled with anti-CD4 (GK1.5) and anti-CD8a (53–6.7) biotinylated antibodies. Purified cells were stained with anti-CD4-APC, anti-CD8a-PB, anti-CD25-PE and anti-CD44-PE-Cy7. In all cases, after labeling, cells were fixed with PFA 2% for 20 min at room temperature and permeabilized in Perm buffer (PBS-2% FCS-0.5% saponin). Then, the cells were stained for 1 h with EZH2 or H3K27me3 primary antibody in Perm buffer, washed, stained for 1 h with anti-rabbit-A488 secondary antibody and washed again before analysis. Data acquisition was performed using a Gallios Beckman Coulter flow cytometer, and data analysis was performed using FlowJo software (TreeStar Inc., OR, USA).

To evaluate EZH2 and H3K27me3 levels during T lymphopoiesis, the median intensity of EZH2 or H3K27me3 staining for each maturation stage was normalized over the median intensity of EZH2 or H3K27me3 staining at the DP stage.

To evaluate the correlation between EZH2 and H3K27me3 staining at each maturation stage, the median intensity of EZH2 staining was plotted against the median intensity of H3K27me3 staining for each mouse, and then bilateral Pearson correlation tests were performed.

### Flow cell imaging

A total of 2 × 10^6^ cells were stained for flow cytometry with the following modifications. A mix of antibodies was anti-CD4-APC, anti-CD8a-FITC, anti-CD25-PE and anti-CD44-PE-Cy7 or anti-CD4-APC, anti-CD8a-FITC, anti-CD3- PE-Cy7 and anti-TCRβ-PE. After EZH2 staining, secondary antibody staining was performed with anti-rabbit CF750. Then, the cells were washed and stained for 5 min with Hoechst 33,342 diluted 1/18,000 in Perm buffer, washed again and finally resuspended in 60 µL of PBS before analysis. A total of 10^5^ events were acquired from the ImageStream × multispectral imaging flow cytometer (Amnis Corporation/EMD Millipore) using the Inspire™ instrument control application (Amnis Corporation). The instrument acquires 12 images in 3 different modes (brightfield, darkfield and fluorescence). The light is collected and projected on 2 times delay integration–charge-coupled device camera. The magnification was 60 × with a higher numerical aperture of 0.90 that generates the lowest pixel resolution (0.33 µm/pixel). Images were acquired with a normal depth of field, providing a cross-sectional image of the cell with a 2.5 µm depth of focus. For these experiments, an SSC laser (785 nm) was not used, so the fluorescence associated with PC7 could be collected. The 405 nm, 488 nm, 561 nm and 658 nm solid-state lasers were used at 80 mW, 100 mW, 100 mW and 120 mW, respectively. Channels 1, 2, 3, 6, 7, 11 and 12 were used to collect fluorescence images of brightfield, FITC, PE, PC7, Hoechst, APC and CF750, respectively. Image data were processed and analyzed using the IDEAS (Image Data Exploration and Analysis software—Amnis Corporation) application. A compensation matrix for spectral spillover was calculated using the single stained controls and applied to experimental files. A mask representing the nucleus was defined thanks to Hoechst 33,342 fluorescence and the morphology mask. The whole cell mask was defined thanks to the brightfield and object masks. Another mask representing the cytoplasmic area was defined by subtracting the nucleus mask from the whole mask.

The similarity feature, corresponding to the log transformed Pearson’s correlation coefficient (PCC, ρ), was determined using the formula ρ = ∑_i_(x_i_ − X)(y_i_ − Y)/√∑_i_(x_i_ − X)^2^ ∑_j_(y_j_ − Y)^2^. The log transformed Pearson’s correlation coefficient is a measure of the degree to which two images are linearly correlated within a masked region.

We also determined the similarity score using the formula: similarity score = ln[(1 + ρ)/(1 − ρ)]. The similarity score is a log transformation of PCC to increase the dynamic range of the function and to roughly give a Gaussian distribution.

### Cell lines stimulation

Cells lines at 13.5 × 10^6^ cells/ml were starved overnight in IMDM-2% FCS. On the stimulation day, 2 × 10^5^ cells were placed on Polysine slides (Thermo Fisher) and incubated for 15 min at 37 °C and 5% CO_2_. During this time, the Dynabeads™ mouse T-activator CD3/CD28 was prepared following the manufacturer’s instructions. Five microliters of Dynabeads was resuspended in 70 µl of IMDM-2% FCS and placed on the cells. Slides were then placed on a magnet for 30 s to rapidly deposit beads on cells and incubated for different durations at 37 °C and 5% CO_2_. After stimulation, cells were fixed for 15 min at room temperature in Microtubule Stabilization Buffer (MTSB: PIPES 80 mM pH 6.8, EGTA 5 mM, MgCl_2_ 1 mM, Triton X100 0.2%) supplemented with PFA 2%, and immunofluorescence was performed.

### Immunofluorescence

A total of 2 × 10^5^ cells of the two cell lines or 5 × 10^5^ thymocytes were plated on Polysine slides, incubated for 15 min at 37 °C and 5% CO_2_ and fixed for 15 min at room temperature in MTSB supplemented with PFA 2%. Cells were stained with primary anti-EZH2 (1/100) and anti-α-tubulin (1/250) antibodies or with primary anti-α-tubulin (1/250) and anti-γ-tubulin (1/500) antibodies in MTSB for 1 h, then with secondary anti-rabbit-A488 (1/250) and anti-rat-A546 (1/250) antibodies or with anti-rat-A546 (1/250) and anti-mouse-DL488 (1/300) diluted in MTSB for 1 h. Nuclei were stained for 5 min with Hoechst 33,342 diluted 1/10,000 in MTSB.

### Immunofluorescence acquisition and analysis

Images were acquired using an SP5 confocal microscope (Leica) with a 63 × oil immersion objective for cell lines or a 100 × oil immersion objective for thymocytes. In both cases, 2 × numerical zoom was applied. For MTOC polarization analysis, z-stack images (0.17 µm spacing) were acquired. Image processing was performed using Fiji (ImageJ) software. For each cell-bead couple, the MTOC polarity index was calculated as described in Obino et al.^37^ with some modifications. Briefly, the geometrical center of the bead (B_c_) was manually selected. For center of mass of each cell (Cell _CM_) positioning, α-tubulin z-stack was projected (SUM slice), and then Cell _CM_ was calculated. The MTOC position was manually selected for each cell-bead couple in the z-stack using γ-tubulin or α-tubulin. In this second case, the MTOC position was estimated using the center of the tubulin aster. The position of MTOC orthogonal projection (MTOC_proj_) on the B_c_–Cell_CM_ vector and the length of the B_c_–MTOC_proj_ vector were calculated. Length of B_c_–Cell_CM_ vector was also calculated. The polarization index corresponds to B_c_–MTOC_proj_ length divided by B_c_–Cell_CM_ length. The median polarity index for each time point was calculated. The mean and standard deviation of three independent experiments were then calculated.

### EZH2 staining measurement along MTOC and microtubules

Staining intensity measurements were performed using Fiji (ImageJ, version 1.52p) software. For each cell-bead couple, z-stack images of MTOC EZH2 costaining and microtubule EZH2 costaining were selected and added using the SUM slide function. From the obtained images, we selected regions of EZH2 and α-tubulin costaining and measured the integrated density as well as the area of the selected regions. On each image, we evaluated the background of EZH2 staining via the measurement of three different regions without EZH2 microtubule colocalization in the cell. Then, for colocalization staining and for background evaluation, the mean EZH2 intensity was determined by dividing the integrated density value by the area of the region. Finally, for each cell, microtubule EZH2 staining was normalized over the EZH2 background. The mean ± SD of three independent experiments was then calculated.

### EZH2 inhibition

Cell lines at 1.5 × 10^6^ cells/ml were starved overnight in IMDM-2% FCS. On the stimulation day, 2.5 × 10^6^ cells/ml were mixed with 5 µM EZH2 inhibitor III SAH-EZH2 (the most specific of both inhibitors) or 5 µM astemizole and incubated for 1 h. Cells were then plated on Polysine slides and stimulated as described above. During all steps, EZH2 inhibition was maintained.

### Co-immunoprecipitation and western blot

For one immunoprecipitation, 20 × 10^6^ SCIET27 cells were lysed in 350 µL of HEPES buffer (50 mM HEPES pH 7.6, 75 mM NaCl, 1 mM MgCl_2_, 1 mM EGTA, 1 mM EDTA, 1% NP-40) during 30 min on ice, representing approximately 1200 µg of proteins for one immunoprecipitation. After a centrifugation at 16000xg for 10 min at 4 °C, the supernatant was collected. Immunoprecipitation was performed by added 2 µg of antibody (or 2µL of EZH2 antibody) to the supernatant and incubation overnight at 4 °C under rotation. Then, 25µL of Dynabeads™ protein A were added, and the complex was immunoprecipitated by incubation at room temperature during 20 min. Beads were washed two times in PBS 0.1% NP-40 and two times in PBS 0.05% NP-40. Finally, the complex was eluated in 1 volume of HEPES buffer and 1 volume of Laemmli with an incubation of 5 min at 95 °C, and the western blot was performed. Briefly, proteins were separated on a 10% SDS–polyacrylamide gel and transferred to PVDF (polyvinylidene difluoride) membranes (Amersham, Buckinghamshire, UK). Primary antibodies were incubated overnight at 4 °C; corresponding secondary antibodies coupled to HRP were incubated for 1 h at room temperature. Blots were stripped and reprobed as necessary. Pierce ECL western blotting substrate (ThermoFisher) was used for immunodetection and signals were detected by chemiluminescence using the Fusion FX7 camera (Vilbert-Lourmat, France).

### Statistical analysis

Chi^2^ tests were used for percentage comparisons. For the analysis of IFC, Friedman and pairwise Wilcoxon signed-rank post hoc tests were performed using the Anastats website (http://www.anastats.fr/outils.php). For flow cell imaging, the Friedman test and its post hoc associated test were performed using the Anastats website. For flow cytometry and polarization index analyses, *StatView* software (SAS Institute Inc., Cary, NC, USA) was used. Homogeneity of variance was determined using the Levene test, and the normality of distribution was determined using the Kolmogorov–Smirnov test. When homogenous variances and distributions were observed, one-way ANOVA analyses were performed followed by PLSD Fisher post hoc tests. When variance and/or distribution were not homogeneous, Kruskal–Wallis nonparametric tests were performed followed by Dunn’s post hoc tests. For correlation analyses of H3K27me3 and EZH2 intensity variation during T lymphopoiesis, bilateral Pearson correlation tests were performed using the Anastats website. For correlation analyses of polarization indices calculated using α- or γ-tubulin, normality of distribution was tested using the Shapiro–Wilk test. Then, bilateral Spearman correlation tests were performed. If the normality of the distribution was not validated, a bilateral Pearson correlation test was performed. Tests were performed using the Anastats website. *P* values < 0.05 indicate significance. All results are shown as the mean ± standard deviation (SD).

## Supplementary Information

Supplementary Information.
