# Supplementary material for: Link between the EZH2 noncanonical pathway and microtubule organization center polarization during early T lymphopoiesis
Source: Sci Rep. 2022 Mar 7;12:3655. doi: 10.1038/s41598-022-07684-5 (PMC8901749; doi:10.1038/s41598-022-07684-5)

Link between the EZH2 noncanonical pathway and microtubule organization center polarization during early T lymphopoiesis

Frederique Deshayes[1], Magalie Fradet[2], Sandra Kaminski[3], Mireille Viguier[1], Jean-Pol Frippiat[3]* and Stephanie Ghislin[3]*

[1] Université de Paris, Unité de Biologie Fonctionnelle et Adaptative, Paris, France.

[2] Université de Paris, CNRS, Institut Jacques Monod, F-75006, Paris, France. Plateforme ImagoSeine-Cytométrie en flux.

[3] EA 7300, Stress Immunity Pathogens Laboratory, Faculty of Medicine, Lorraine University, 9 avenue de la forêt de Haye, F-54500 Vandœuvre-lès-Nancy, France.

*Jean-Pol Frippiat and Stephanie Ghislin jointly supervised this work

Supplemental figure legends

Supplemental Figure S1

EZH2 subcellular localization changes during T lymphopoiesis. EZH2 subcellular localization was analyzed by flow cytometry imaging. (**A**, **B**, **C**) Thymocytes were identified using CD4, CD8, CD3, TCR, CD25 and CD44 staining. Five subpopulations of DN cells were identified according to CD44 and CD25 staining, *i.e.*, DN1 $CD44_{high}$ ($CD44^{high}/CD25^{-}$), DN1 $CD44_{low}$ ($CD44^{low}/CD25^{-}$), DN2 ($CD44^{low}/CD25^{+}$), DN3 ($CD44^{-}/CD25^{+}$) and DN4 ($CD44^{-}$ $CD25^{-}$). (**D**) Dot plots representing similarity score versus EZH2 fluorescence intensity for each subpopulation. Number at bottom left indicated number of cells in the population. It is to note that analysis was considered significant with at least 50 cells in a gate.

Supplemental Figure S2

EZH2, EED, Suz12 and H3K27me3 are differently regulated during T lymphopoiesis. EZH2, EED, Suz12 and H3K27me3 levels were evaluated at each stages of T lymphopoiesis by flow cytometry. Thymocytes were identified using CD4, CD8, CD3, TCR, CD25 and CD44 staining. Five subpopulations of DN cells were identified according to CD44 and CD25 staining, *i.e.*, DN1 $CD44_{high}$ ($CD44^{high}/CD25^{-}$), DN1 $CD44_{low}$ ($CD44^{low}/CD25^{-}$), DN2 ($CD44^{low}/CD25^{+}$), DN3 ($CD44^{-}/CD25^{+}$) and DN4 ($CD44^{-}$ $CD25^{-}$). For each stage, dot plots representing SS-C versus fluorescence intensity of EZH2 (**A**), H3K27me3 (**B**), EED (**C**) and Suz (**D**) are showing. Dot plots correspond to one representative mice.

Supplemental Figure S3

Validation of EZH2 antibody specificity. To validate EZH2 antibody specificity, EL4 cell line (cell line which presents best transfection efficiency) was transfected with specific EZH2 siRNA or negative control (scramble) by electroporation in 1SM buffer (5mM KCl; 15mM MgCl2; 120mM Na2HPO4/NaH2PO4 pH7.2; 25mM Sodium Succinate; 25mM Manitol)

supplemented with 5% BSA (Chikaybam et al., 2013) using Nucleofector II from Lonza. For western blot different concentrations of EZH2 siRNA has been tested. For immunofluorescence EL4 cells were transfected with 4.5mM of siRNA. After 24h incubation, immunofluorescence (**A**) and western blot (**B**) were performed. For western blot, EZH2 antibody used correspond to the one used in flow cytometry and immunofluorescence all along the study, α-tubulin was used as loading control. For imaging EL4 cells same setting has been used between conditions. Histogram represents the mean of three independent experiments ± SD, and ANOVA tests were performed followed by *post hoc* PLSD Fisher tests. ** $p<0.01$. (**C**) To validate existence of EZH2 and tubulin network colocalization, a second EZH2 antibody (from Merck Millipore 07-689) was tested on thymocytes.

Supplemental Figure S4

Examples of γ-tubulin, α-tubulin and EZH2 staining in SCB29 and SCIET27 cell lines. (**A**) SCB29 (upper panel) and SCIET27 (lower panel) cells were stimulated with the Dynabeads™ mouse T-activator CD3/CD28 for 5- or 30-min. Immunofluorescence was performed, and α- and γ-tubulin were stained red and green, respectively. Nuclear position was determined using Hoechst 33342, and bead position was determined using brightfield (BF) staining and reported in each channel. Images correspond to one representative observation of three independent experiments. (**B**) Examples of EZH2 staining observed along the microtubule network. EZH2 was stained green, and α-tubulin was stained red. For each type, two cells are presented.

Supplemental Figure S1

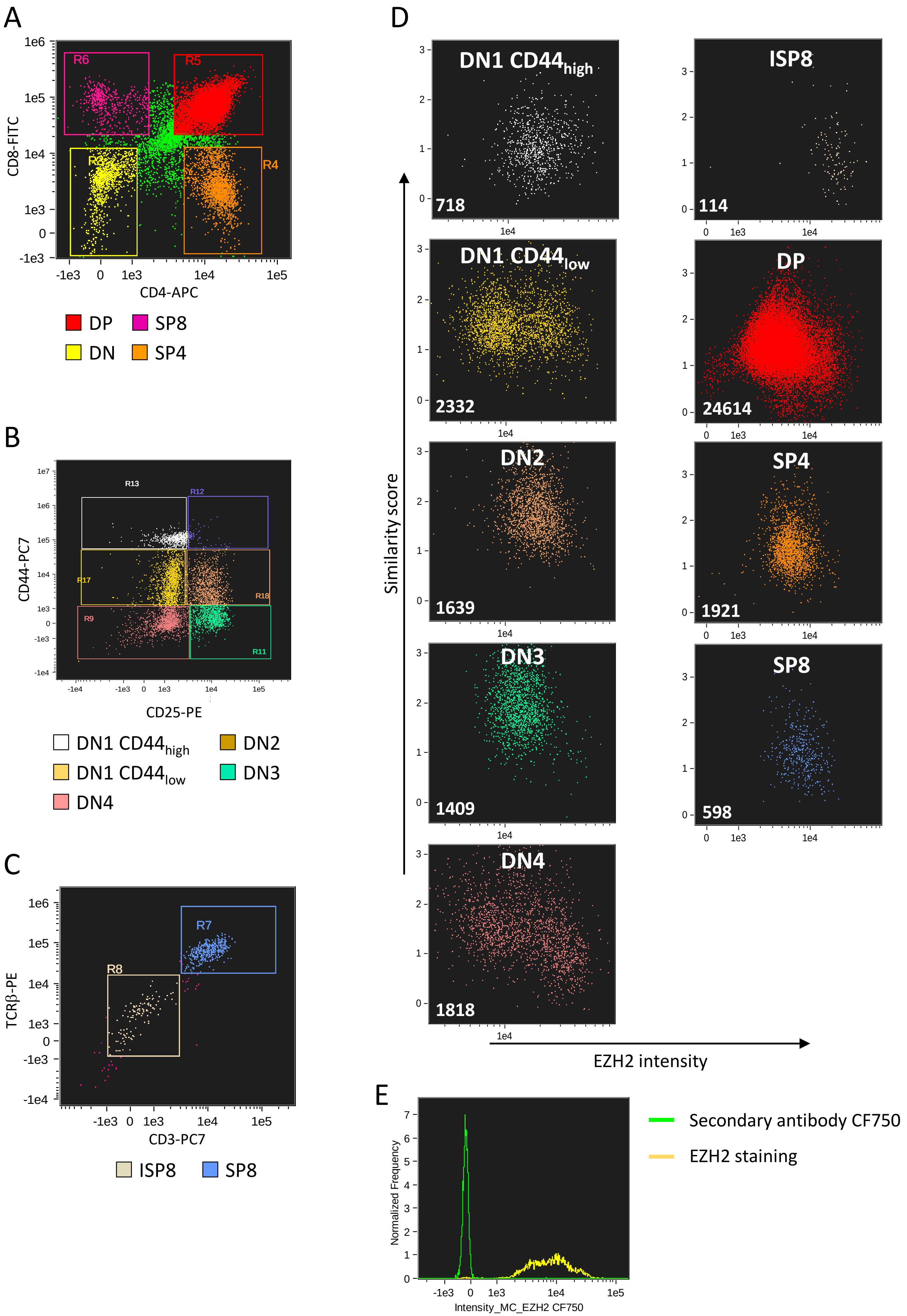

Supplemental Figure S2

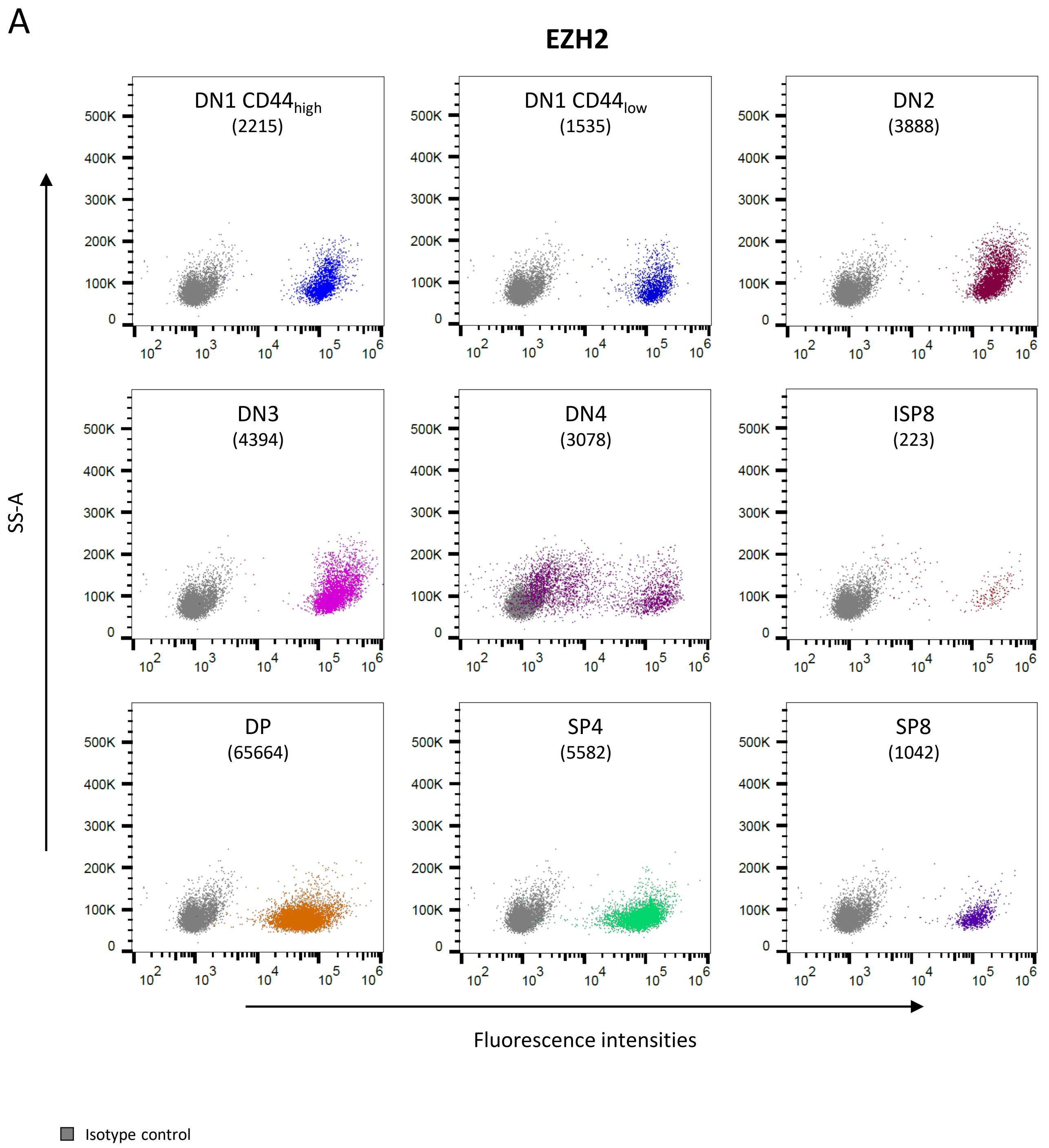

Supplemental Figure S2

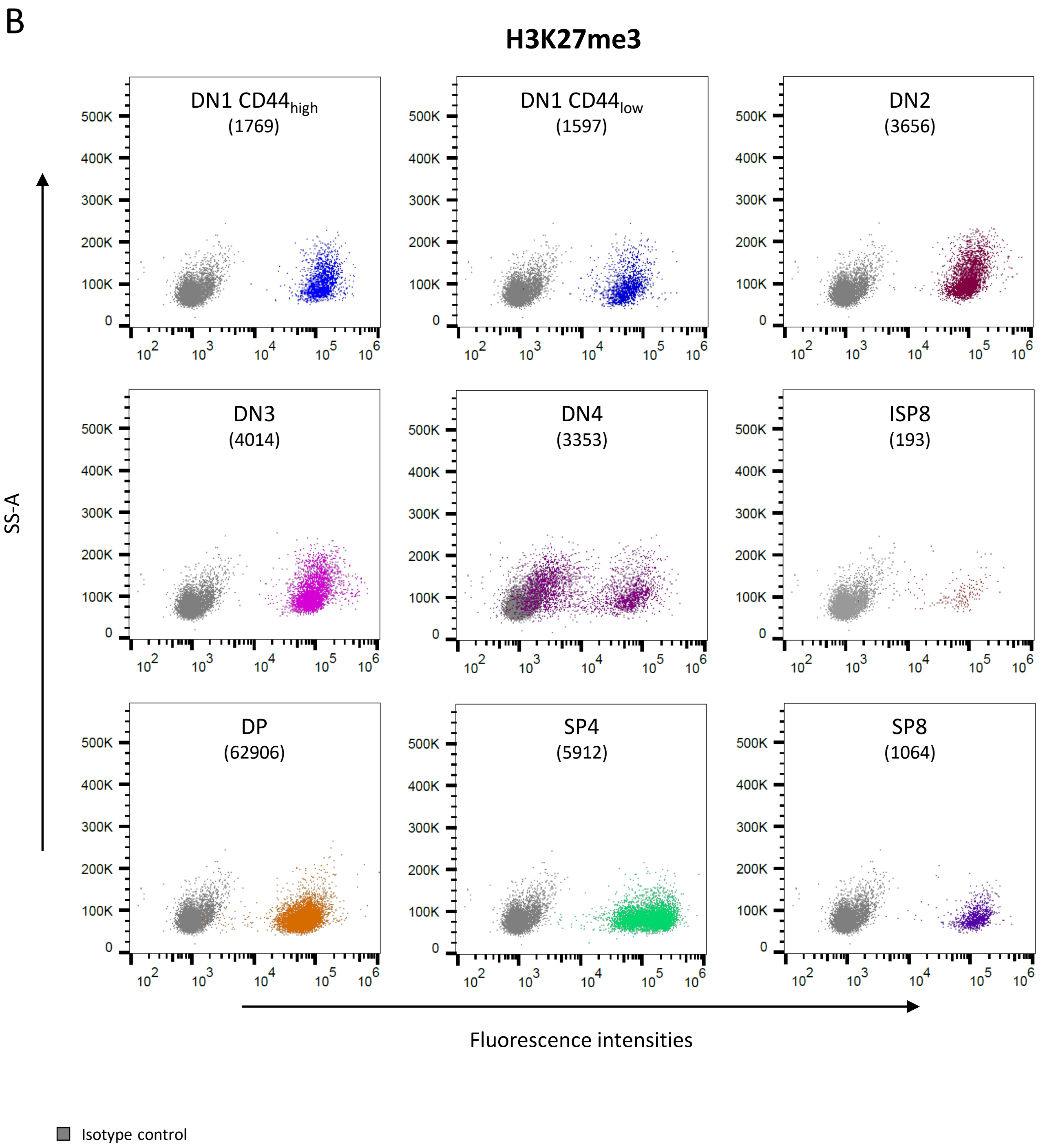

Supplemental Figure S2

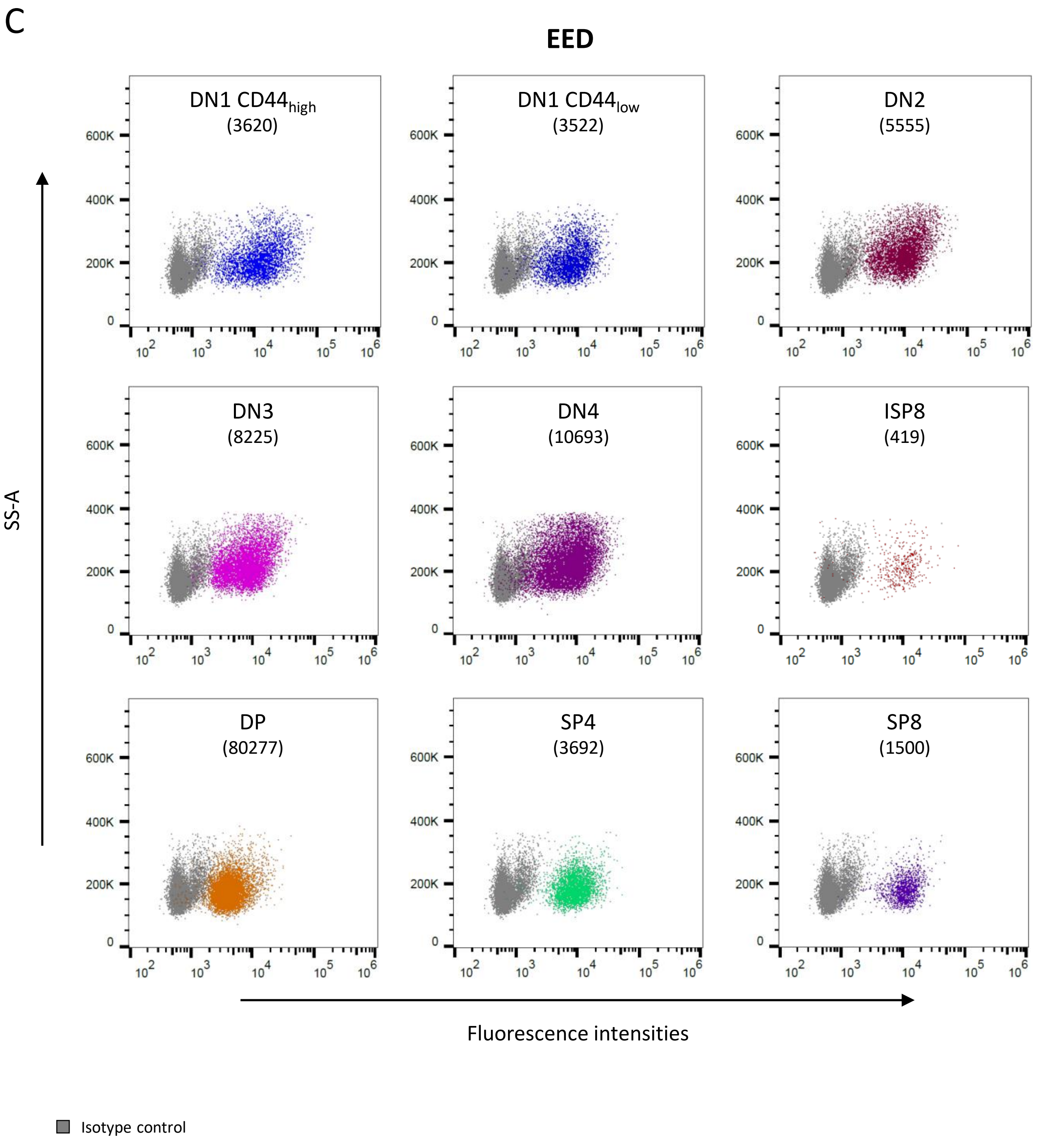

Supplemental Figure S2

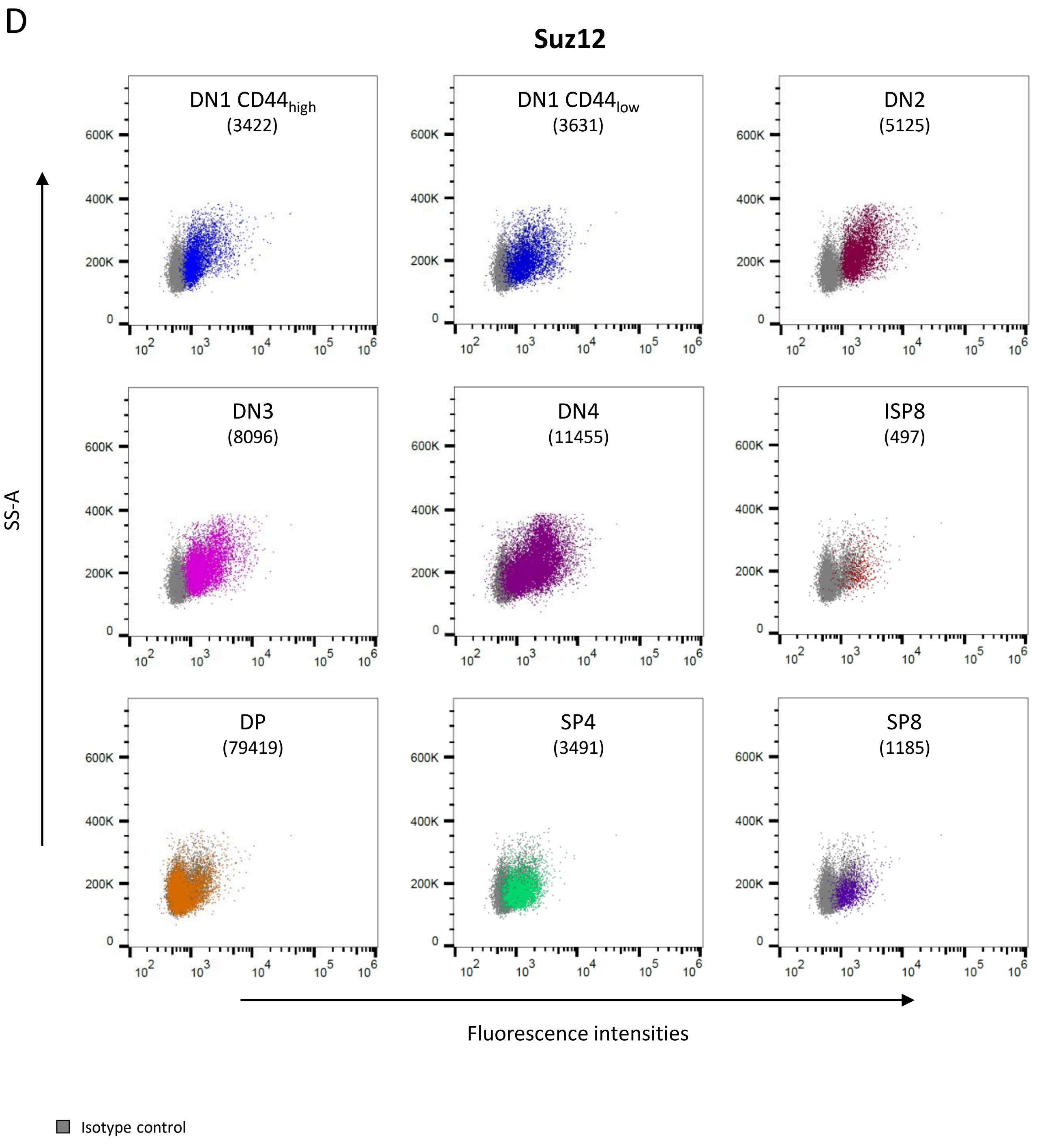

Supplemental Figure S3

A

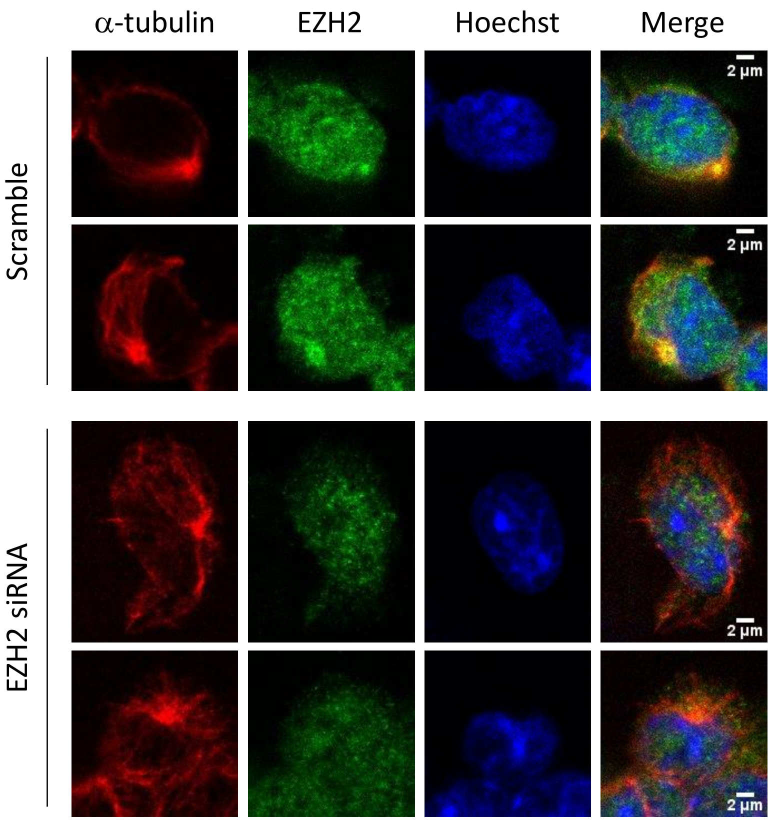

B

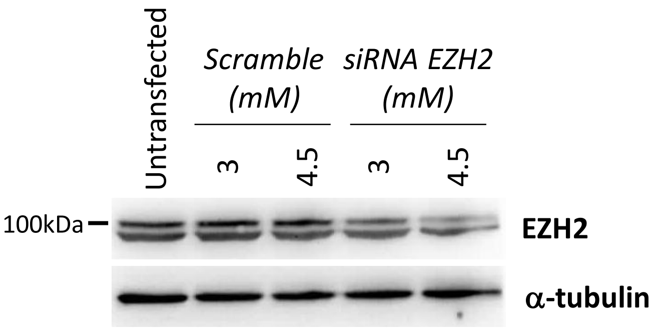

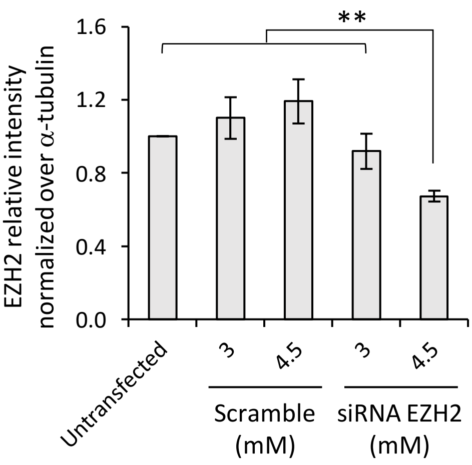

p_{ANOVA}<0.001***

C

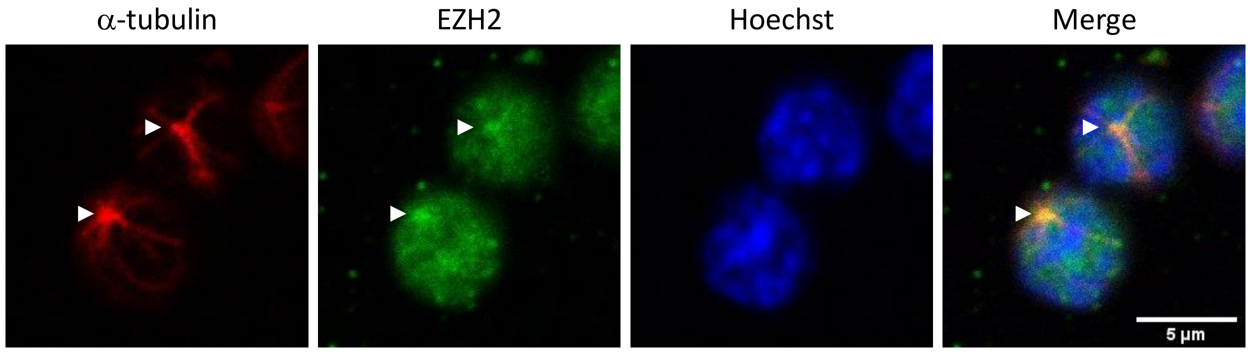

Supplemental Figure S4

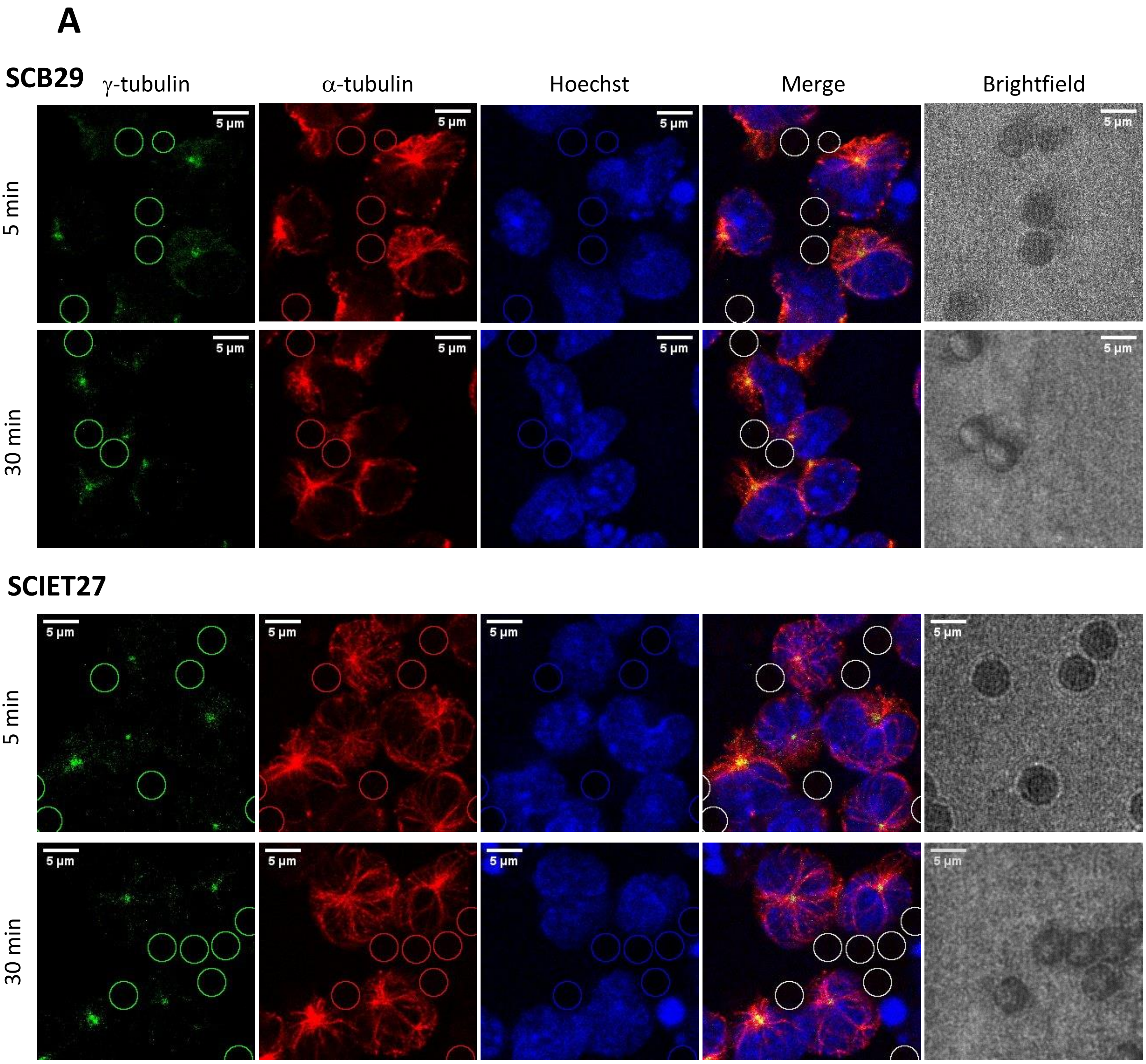

Supplemental Figure S4

B

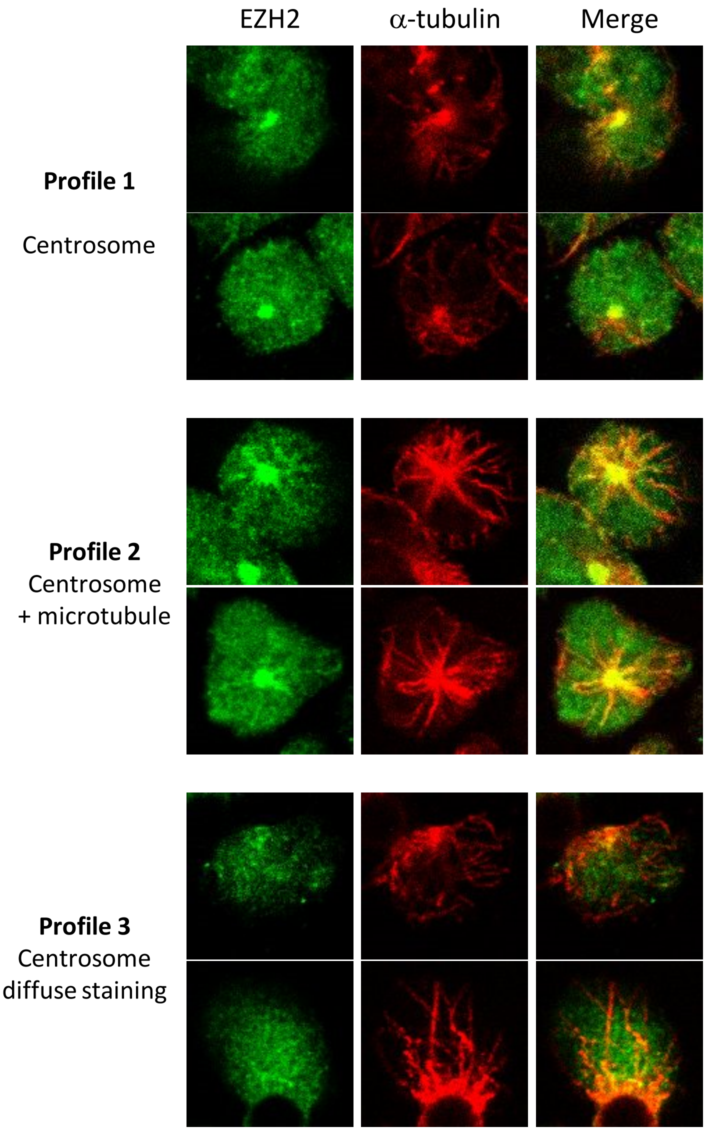

Supplemental Figure S5

A

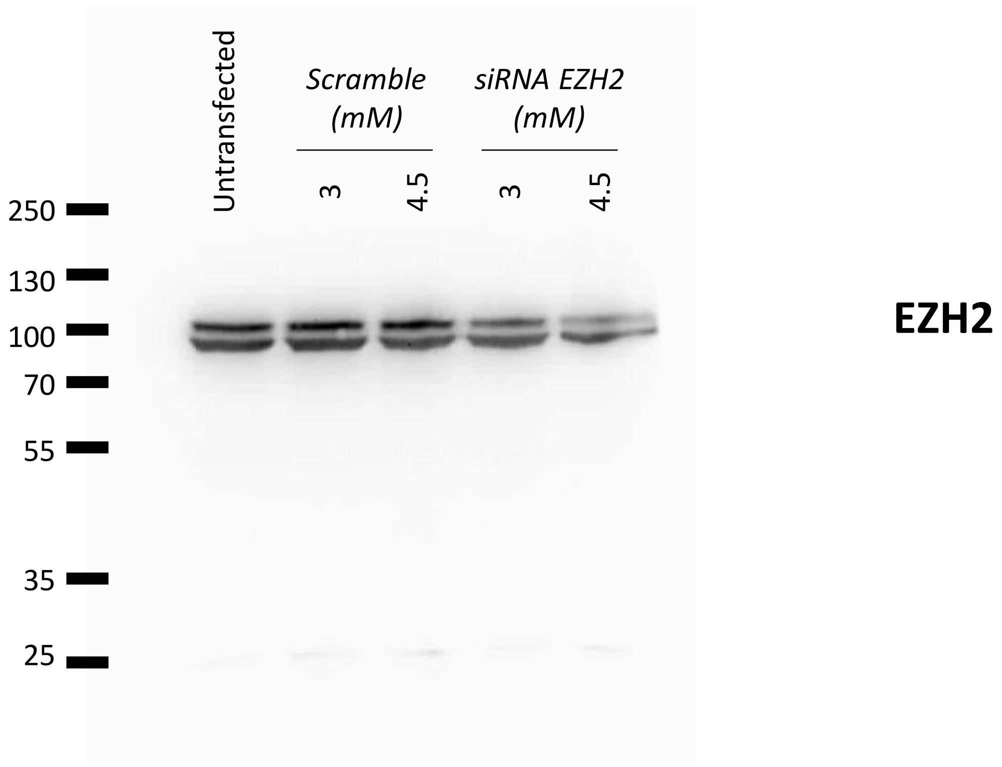

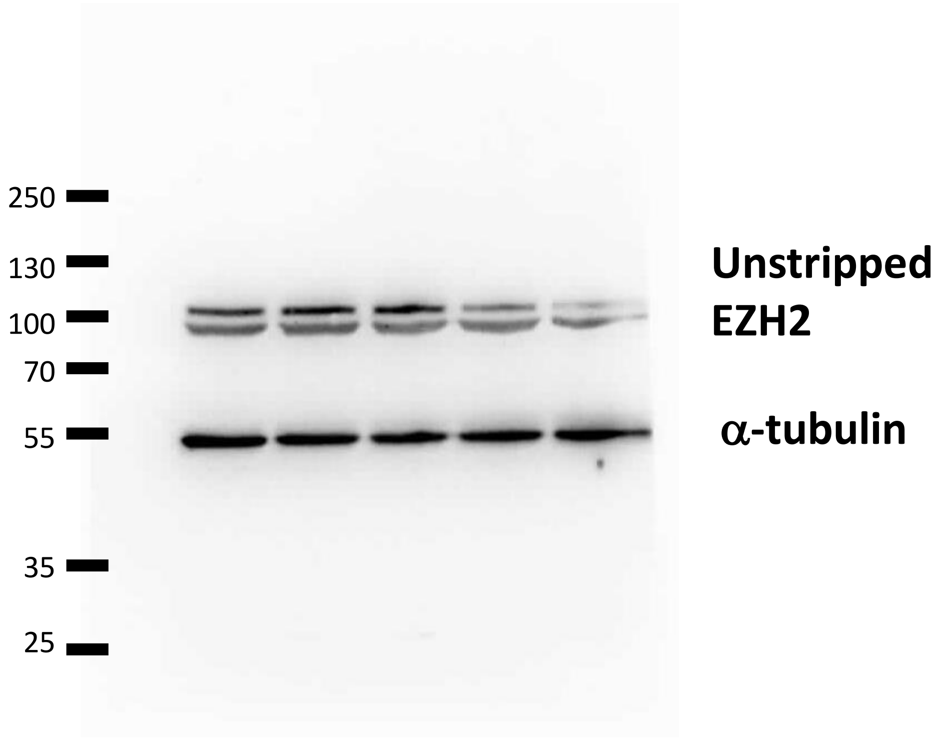

Supplemental Figure S5

B

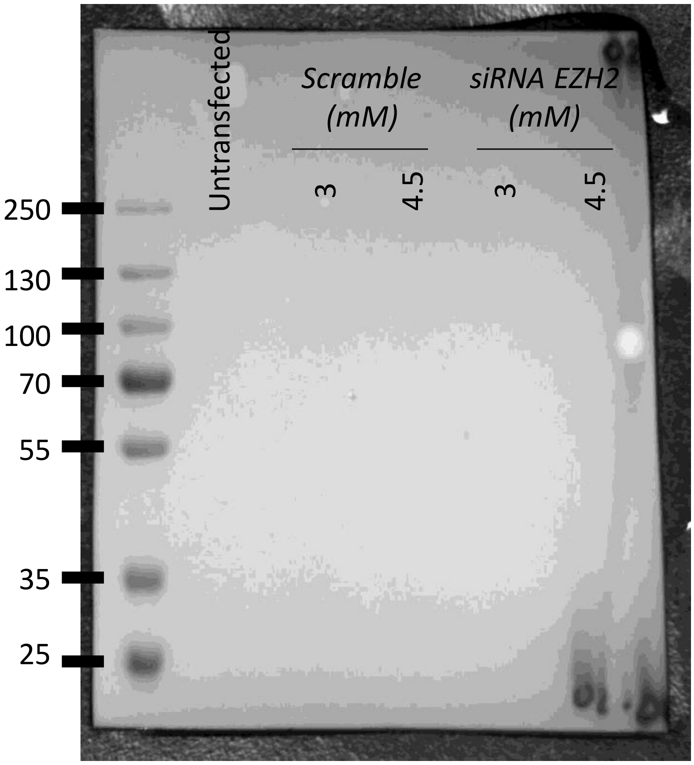

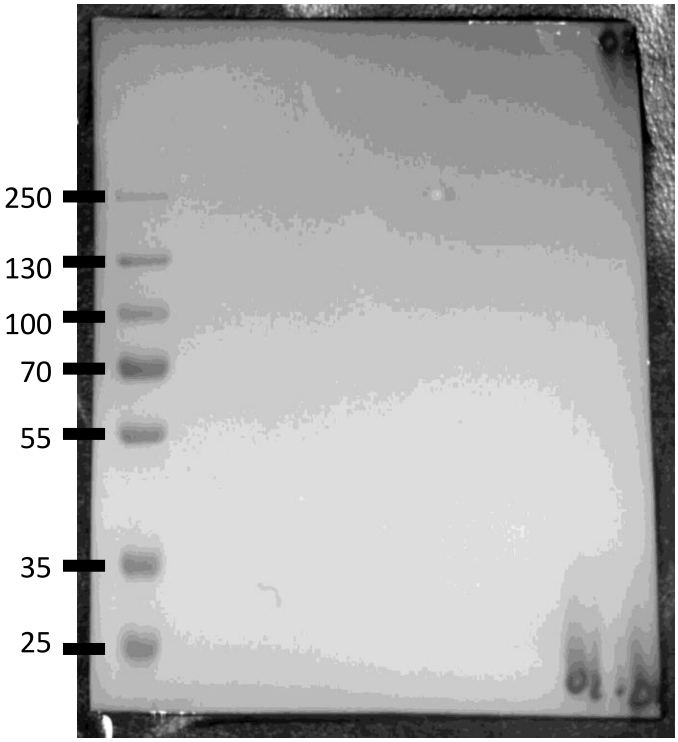

Supplemental Figure S6

A

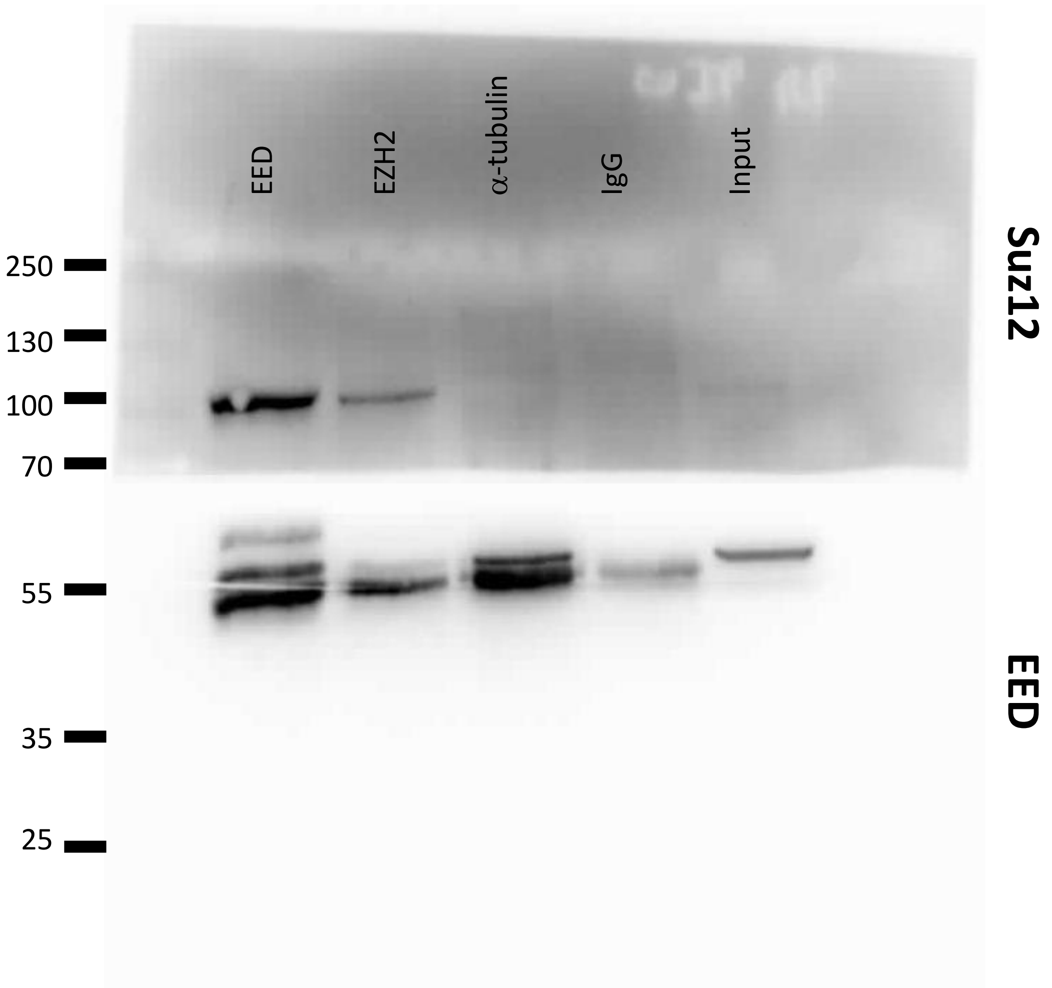

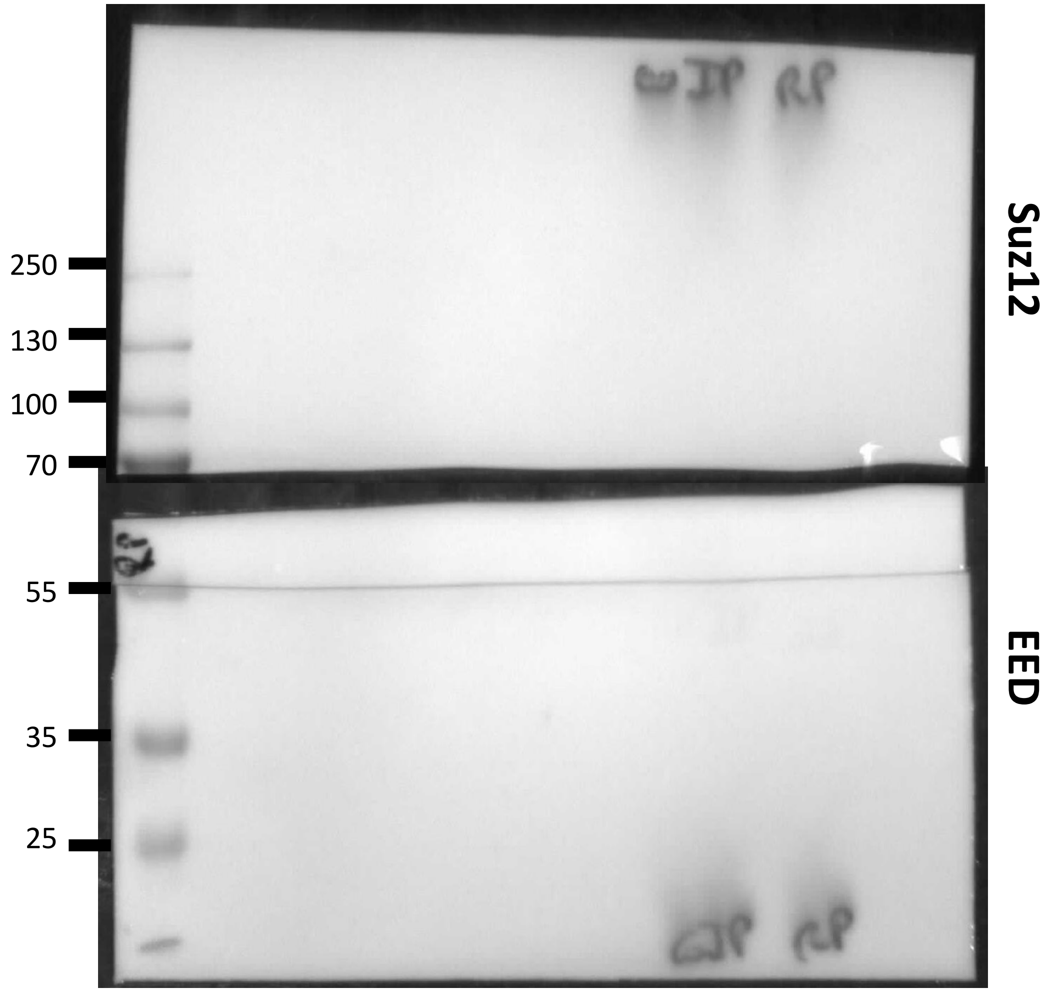

Supplemental Figure S6

B

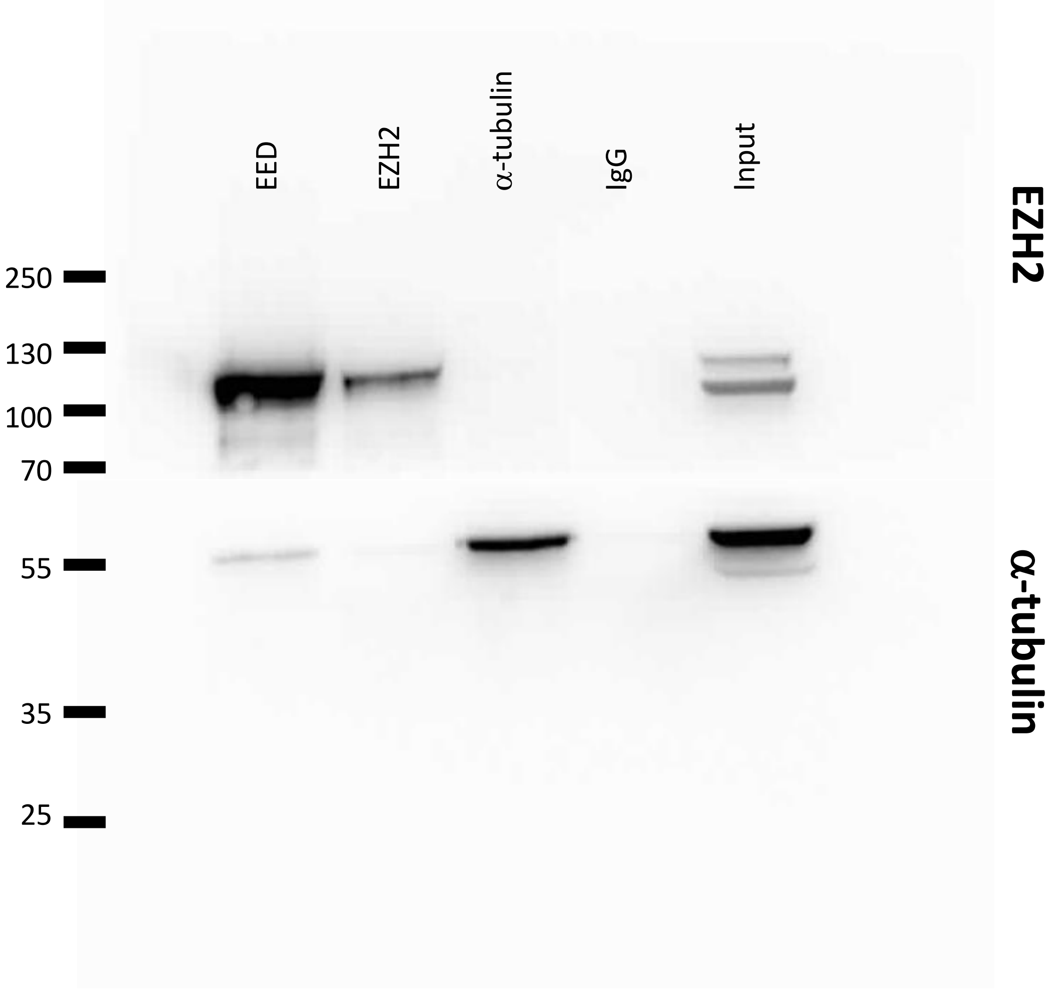

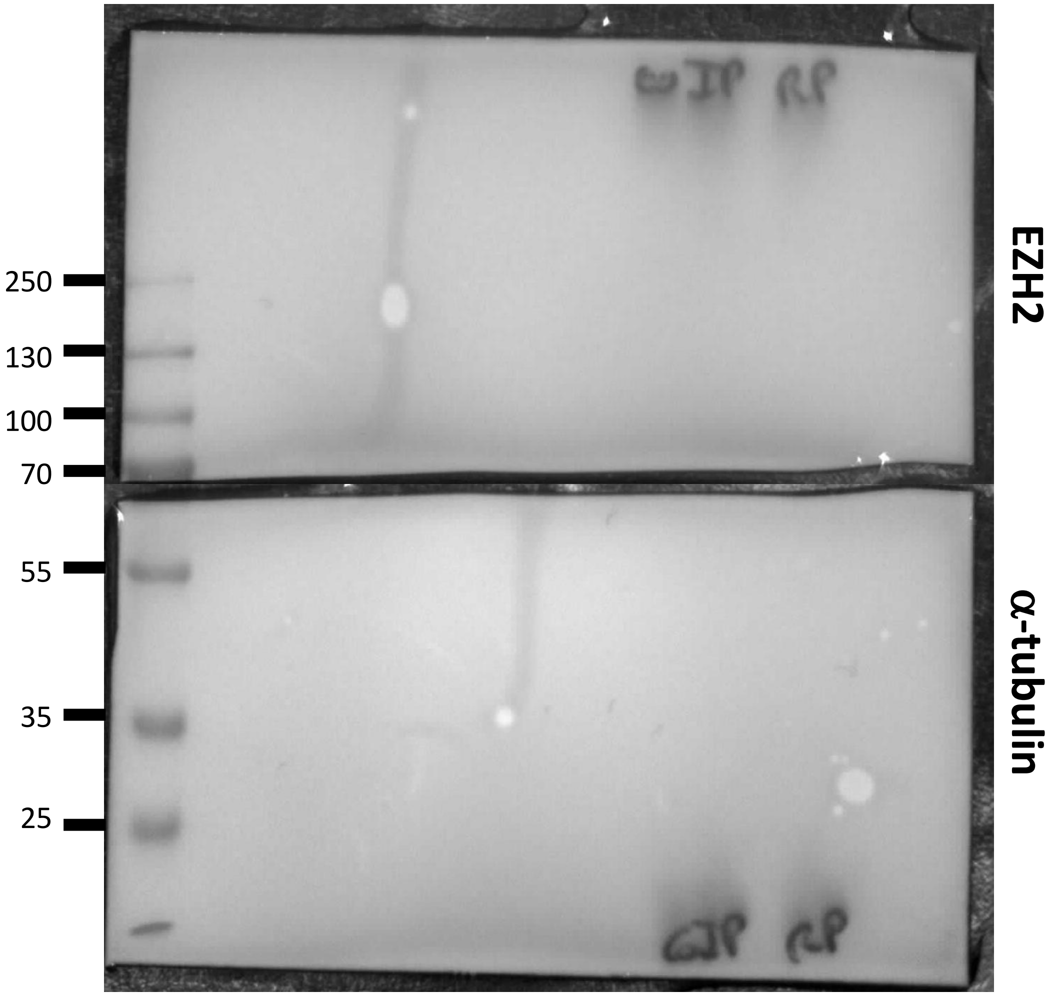

Supplemental Figure S6

C

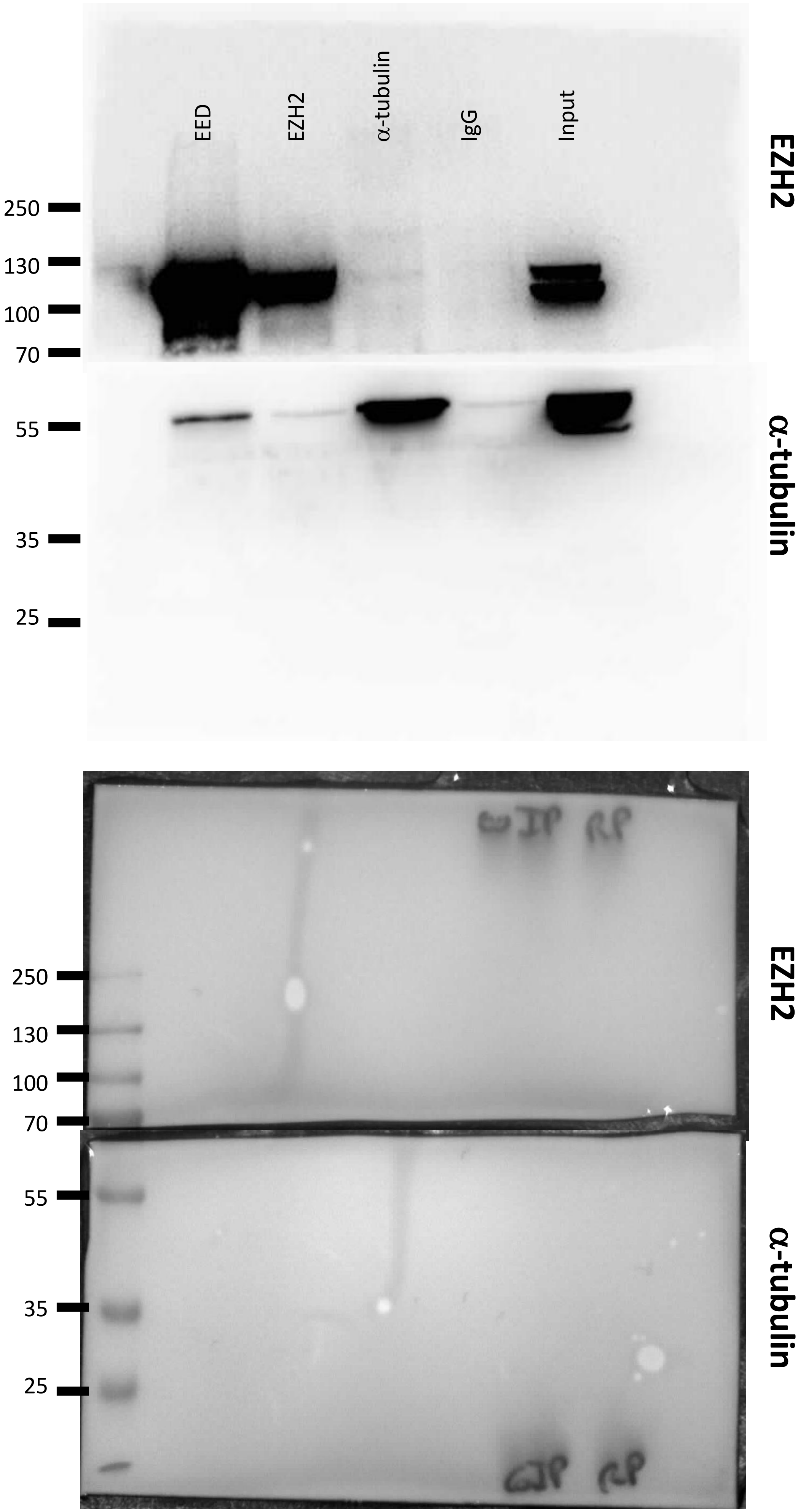

Supplement: Supplementary file 1 — Supplementary Information. [file 41598_2022_7684_MOESM1_ESM.pdf]
